# Selection of nanobodies with broad neutralizing potential against primary HIV-1 strains using soluble subtype C gp140 envelope trimers

**DOI:** 10.1038/s41598-017-08273-7

**Published:** 2017-08-21

**Authors:** Kathrin Koch, Sarah Kalusche, Jonathan L. Torres, Robyn L. Stanfield, Welbeck Danquah, Kamal Khazanehdari, Hagen von Briesen, Eric R. Geertsma, Ian A. Wilson, Ulrich Wernery, Friedrich Koch-Nolte, Andrew B. Ward, Ursula Dietrich

**Affiliations:** 1 Georg-Speyer-Haus, Paul-Ehrlich-Str, 42–44, 60596 Frankfurt, Germany; 2 Department of Integrative Structural and Computational Biology, The Scripps Research Institute, La Jolla California, 92037 USA; 3 Institute of Immunology, University Medical Center Hamburg-Eppendorf, 20246 Hamburg, Germany; 4 Central Veterinary Research Laboratory, Dubai, United Arab Emirates; 5 Fraunhofer Institute for Biomedical Engineering, 66280 Sulzbach, Germany; 6 Institute of Biochemistry, Biocenter, Goethe-University Frankfurt, Frankfurt, Germany

## Abstract

Broadly neutralizing antibodies (bnAbs) against HIV-1 protect from infection and reduce viral load upon therapeutic applications. However no vaccine was able so far to induce bnAbs demanding their expensive biotechnological production. For clinical applications, nanobodies (VHH) derived from heavy chain only antibodies from *Camelidae*, may be better suited due to their small size, high solubility/stability and extensive homology to human VH3 genes. Here we selected broadly neutralizing nanobodies by phage display after immunization of dromedaries with different soluble trimeric envelope proteins derived from HIV-1 subtype C. We identified 25 distinct VHH families binding trimeric Env, of which 6 neutralized heterologous primary isolates of various HIV-1 subtypes in a standardized *in vitro* neutralization assay. The complementary neutralization pattern of two selected VHHs in combination covers 19 out of 21 HIV-1 strains from a standardized panel of epidemiologically relevant HIV-1 subtypes. The CD4 binding site was preferentially targeted by the broadly neutralizing VHHs as determined by competition ELISAs and 3D models of VHH-Env complexes derived from negative stain electron microscopy. The nanobodies identified here are excellent candidates for further preclinical/clinical development for prophylactic and therapeutic applications due to their potency and their complementary neutralization patterns covering the majority of epidemiologically relevant HIV-1 subtypes.

## Introduction

The envelope glycoprotein (Env) of HIV-1 is essential for viral infectivity by mediating virus binding to the CD4 receptor followed by interaction with one of the co-receptors, CCR5 or CXCR4, and finally membrane fusion. As such, Env is targeted by broadly neutralizing antibodies (bnAbs) interfering with HIV-1 entry. The functional Env unit on the surface of the virion is a trimeric spike composed of three heterodimers of the outer envelope glycoprotein gp120 and the transmembrane glycoprotein gp41. Both remain associated in a non-covalent manner after cleavage by furin from the common precursor gp160. The rather unstable and highly glycosylated trimer is further characterized by metastability and extreme conformational flexibility, which is necessary to mediate the consecutive viral entry steps and also allows -besides other evasion mechanisms- protection of important late entry epitopes from neutralizing antibodies^1^. In order to stabilize the Env trimers for antigenic and structural analyses, soluble trimeric gp140 SOSIP constructs have been derived, where the extracellular part of gp41 is covalently linked to gp120 by an engineered disulfide bond (SOS) and an isoleucine to proline mutation (IP) in gp41^2^. These SOSIPs and next generation derivatives^3–5^, have been shown to be good antigenic mimics of the native Env spike in its closed prefusion conformation^4, 6^. Nevertheless, even these immunogens were not able so far to induce a robust bnAb response against heterologous Tier 2 viruses upon vaccination^7–9^; at best autologous tier 2 responses have been observed in animal models^10, 11^.

In contrast to the results obtained with HIV envelope vaccines, very effective bnAbs against HIV have been identified in recent years from a subset of individuals chronically infected with HIV-1 favored by the development of sensitive techniques for antigen (Env)-specific B-cell sorting^12–19^. Furthermore, administration of such bnAbs led to the control of viraemia and prevention of infection in humanized mice^20, 21^ and non-human primates^22, 23^. Ultimately, a recent phase I clinical trial with single doses of the bnAb 3BNC117 resulted in reduced viremia^24^ and enhanced immunity in HIV-1-infected individuals^25, 26^ as well as in delayed viral rebound after treatment interruptions in a phase 2a clinical trial^27^. These studies emphasize the prominent role that bnAbs play in prevention and treatment of HIV-1 infection and prove that direct administration of bnAbs is an option for clinical settings in the absence of a suitable Env immunogen able to induce bnAbs upon vaccination. However, for therapeutic and prophylactic applications, nanobodies or VHH derived from *Camelidae* may be better suited due to their small size and favorable physicochemical properties^28^. VHHs correspond to the antigen-binding domain of heavy-chain only antibodies (hcAbs) that, besides conventional IgGs, occur in all members of the *Camelidae*. HcAbs lack the CH1 domain and consequently a paired light chain leaving a single variable antigen recognizing domain, the VHH^29, 30^. Besides their small size (15 kDa) these single domain antibodies are characterized by high solubility, stability and refolding properties^28^. Moreover, VHHs show high affinity in the nanomolar range to their target antigens. Notably, VHHs are characterized by long CDR3 loops (average of 17 amino acids (aa) in contrast to human HCDR3 with on average 12–13 aa in length^31^), which is often a feature of bnAbs against HIV-1 and may allow penetration through the highly glycosylated surface of Env into conserved epitopes not accessible to conventional antibodies^32–35^. The fact that VHHs show a high degree of homology with the VH3 subset of human VH genes and their application as modular building blocks make them a useful tool in view of clinical applications^36^.

Previously, HIV-1 Env-specific VHHs have been selected from llamas immunized with different monomeric gp120 and gp140 Env constructs in the group of Robin Weiss. Whereas immunization with monomeric gp120 did not result in the induction of broadly neutralizing nanobodies^37, 38^, immunization with a mixture of trimeric gp140 from subtype B/C and A led to identification of the first very effective broadly neutralizing nanobodies against HIV-1^39–41^. In this study, we generated three soluble trimeric gp140 SOSIP immunogens derived from HIV-1 subtype C, the most prevalent subtype worldwide^42^, and immunized dromedaries, which have a particularly high proportion of hcAbs (75%)^29^. To focus the immune response on HIV-1 strains responsible for transmission, two animals received a mixture of trimeric gp140 SOSIPs derived from recently transmitted HIV-1 subtype C from Zambia and South Africa, respectively^43^. Two additional animals were immunized with a gp140 SOSIP that we generated from an ancestral subtype C consensus sequence^44^ to potentially broaden the immune response. VHH phage libraries were generated from all four immunized animals and screened on the respective autologous SOSIP constructs. Overall, we could select Env-specific nanobodies from 25 families, of which six nanobodies showed potent neutralization of primary heterologous Tier 2 HIV-1 strains from several subtypes in a standardized pseudovirus-based *in vitro* neutralization assay^45, 46^. In particular, the complementary neutralization patterns of two nanobodies covering 19 of 21 primary strains of the cross-subtype standard reference panel, make these nanobodies promising candidates for further development towards prophylactic/therapeutic applications.

## Results

### Production of soluble trimeric subtype C gp140 SOSIP immunogens

**Figure 1:**
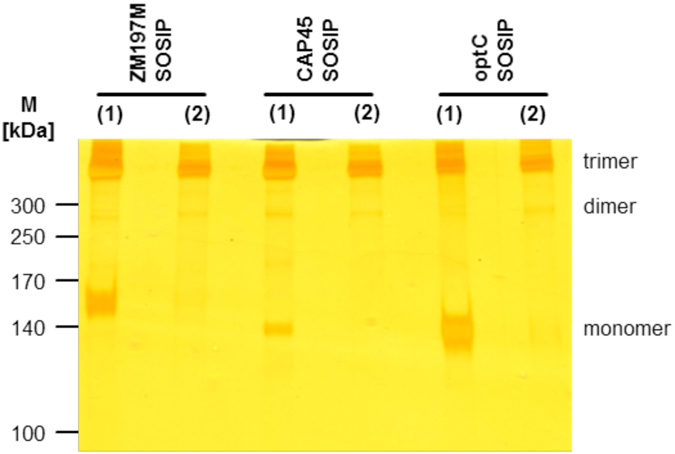
Silver staining of purified ZM197M, CAP45 and optC gp140 SOSIP proteins. Indicated Env gp140 SOSIP proteins are shown after lectin purification (**1**) followed by additional size exclusion and anion exchange chromatography purification steps (**2**). The gel was cropped below the 100 kDa marker and the marker lane was substituted by indicating the molecular weights of the marker proteins. The original gel is shown in Suppl. Figure 1a (1) together with the individual fractions collected after SEC and AEC for the three SOSIPs (2) to (4).

Gp140 SOSIP constructs were generated for HIV-1 subtype C strains, including the recently transmitted ZM197M and CAP45 as well as the ancestral codon-optimized optC, by site-directed mutagenesis from the respective gp160 plasmids. After cloning and transient transfection into 293T cells, soluble SOSIPs were purified from culture supernatants by lectin affinity chromatography, followed by size exclusion (SEC) and anion exchange chromatography (AEC). After SEC and AEC purification, the gp140 monomers visible after lectin purification were mostly eliminated resulting in predominantly trimeric gp140 SOSIPs for all three isolates (Fig. 1, Supplementary Figure S1), although some higher molecular weight aggregates are still visible. The gp140 SOSIPs were further analyzed for binding of a set of known monoclonal Abs (mAbs, see Supplementary Figure S1), both neutralizing and non-neutralizing. Best binding to the optC SOSIP was observed for bnAb VRC01, b12 as well as for CD4-Fc. The neutralizing mAb PGV04 showed intermediate binding; intermediate binding was also seen for the non-neutralizing mAbs F105 and 17b suggesting partially open conformations of the trimeric SOSIPs, which are characteristic for the first generation of SOSIPs. Negative stain electron microscopy of the optC SOSIPs confirmed the presence of partially open trimers attached to a stalk consisting of the MPER regions of the trimer and micelles (Supplementary Figure S1). All three purified subtype C gp140 SOSIPs were used both as immunogens for the vaccination of dromedaries and as target proteins for the selection of HIV-specific nanobodies from immunized animals by the phage display technology.

### Trimeric subtype C GP140 SOSIPs induce specific and neutralizing antibody responses in sera from immunized dromedaries

**Figure 2:**
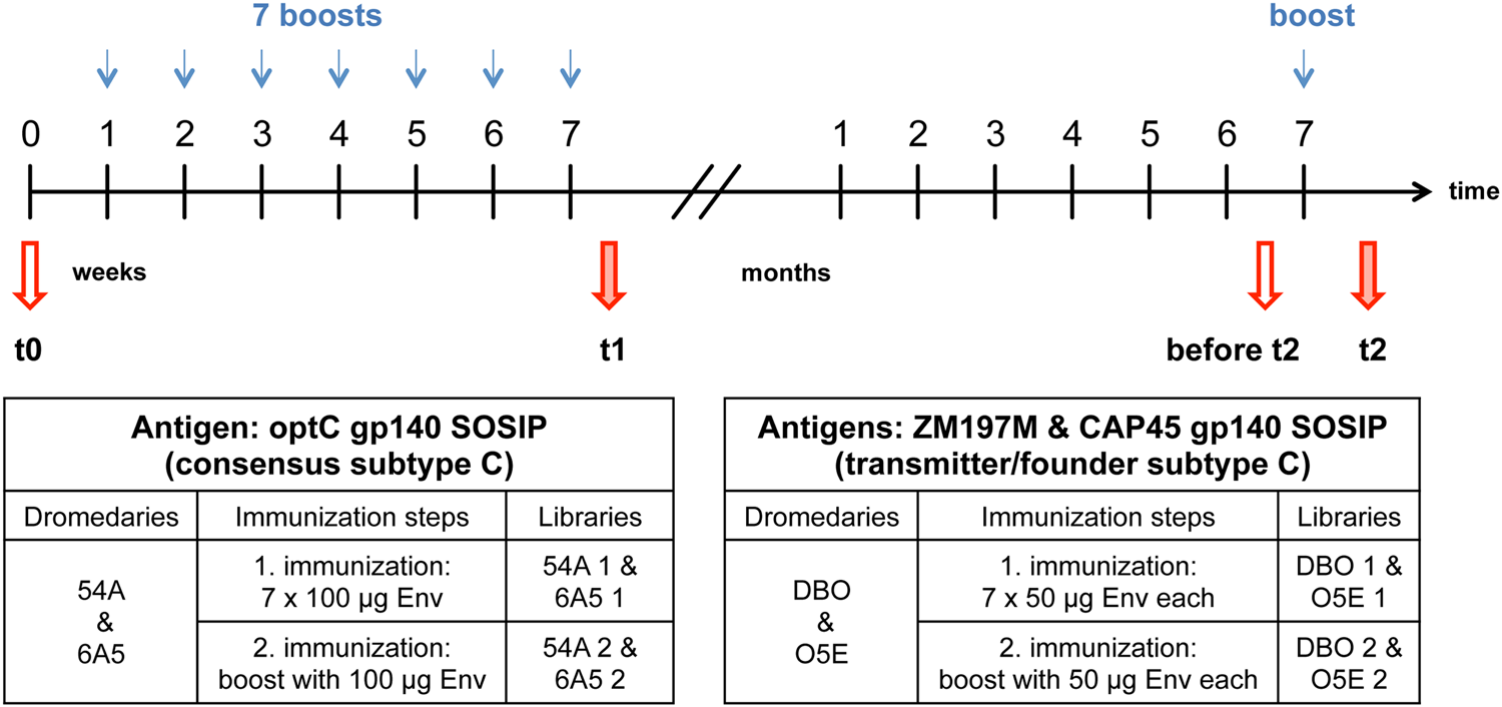
Immunization schedule of dromedaries with subtype C SOSIPs. Animals 54A and 6A5 received optC SOSIP, whereas DBO and O5E received a mixture of ZM197M and CAP45 SOSIPs. The first immunization cycle consisted of 7 weekly protein boosts followed by an additional boost seven months later. Red arrows indicate the timepoints of serum samples used for neutralization assays. Blood samples from timepoints t1 and t2 were also used for the generation of phage displayed nanobody libraries for all 4 animals.

Four dromedaries (54 A, 6A5, DBO and O5E) were immunized with the trimeric subtype C gp140 SOSIPs. All animals were immunized subcutaneously and received a first immunization cycle consisting of seven weekly boosts with trimeric SOSIP, followed by a single boost seven months later (Fig. 2). Two animals (54 A and 6A5) received the optC SOSIP (100 µg per boost) and two animals (DBO and O5E) received a mixture of ZM197M and CAP45 SOSIPs (each 50 µg per boost).

**Figure 3:**
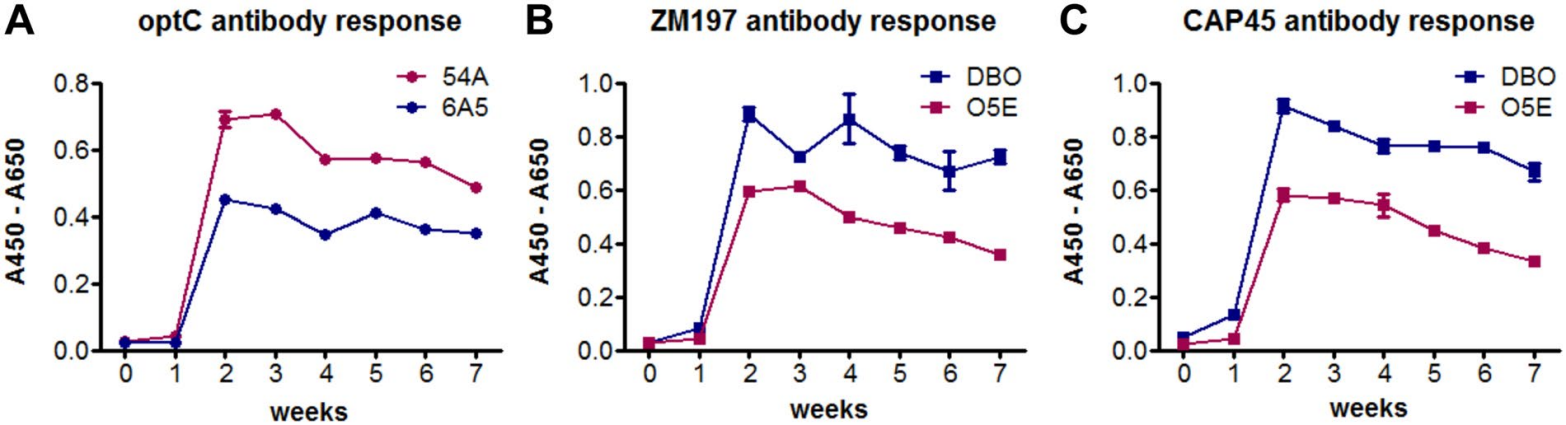
Env-specific antibody response in dromedary sera during the seven immunization boosts of the first immunization cycle. ELISA experiments showing (**A**) optC antibody response in sera from 54A (diluted 1:1,000, open circles) and 6A5 (1:200, black circles), (**B**) ZM197M antibody response in sera from DBO (1:120, open squares) and O5E (1:375, black squares) and (**C**) CAP45 antibody response in sera from DBO (1:120, open squares) and O5E (1:375, black squares). 0: preimmune sera, 1–7: serum samples after the respective immunization boosts. One representative experiment of two is shown. Error bars indicate SEM from triplicates.

The antibody response against the autologous SOSIPs was monitored after every boost during the immunization procedure by enzyme-linked immunosorbent assay (ELISA). All four animals developed a strong Env-specific antibody response one week after the first SOSIP administration (Fig. 3). Interestingly, the antibody response was higher in animal 54 A compared to 6A5, even at a fivefold higher serum dilution. Animal 54A received the ADVAX adjuvant in the first immunization cycle, whereas 6A5 received the GERBU adjuvant. Based on these results, ADVAX was used in all four animals for the second immunization boost. Animals DBO and O5E developed a comparable antibody response against both SOSIP immunogens administered simultaneously. For all animals, the antibody binding response remained high during the 7 injections of the first immunization cycle, after which it declined steadily. After the second single immunization boost (t2), antibody titers increased again for all animals (Supplementary Figure S2).

To initially assess if an HIV-1 neutralizing antibody response was induced in the immunized animals, sera from the four animals were analyzed in a standardized TZM-bl assay using virus particles pseudotyped with the Env proteins from the autologous strains or the heterologous subtype B Bal.26. Immune sera from all four dromedaries neutralized the autologous HIV-1 pseudoviruses, whereas the unrelated MLV control pseudovirus was not neutralized (Table 1, Supplementary Figure S3). Also the heterologous Tier1B Bal.26 strain was neutralized by sera from animals 54A, DBO and O5E (O5E only timepoint t2, Supplementary Figure S4). In most cases, neutralization activity at timepoint t2 (Fig. 2) was superior compared to that of timepoint t1, which was most pronounced for animal O5E immunized with the mixture of ZM197M and CAP45 SOSIPs. As immune sera from animal 54A immunized with optC showed best neutralizing activity, we evaluated these sera further with the heterologous subtype C ZM197M (Tier 1B) and five Tier 2 pseudoviruses representing four different subtypes in the TZM-bl assay. All animal sera from timepoints 1 and 2 from this animal were able to neutralize not only the heterologous isolates matching the subtype of the immunogen (C), but also isolates from heterologous subtypes A, B and G (Table 1, Supplementary Figure S4). These results show that immunization of dromedaries with different soluble trimeric subtype C SOSIPs resulted in the induction of an HIV-specific neutralizing activity in the immune sera against heterologous isolates from different clades.

**Table 1:** Assessment of neutralizing activity (ID_50_)^1^ against HIV-1 in dromedaries’ sera.

All sera against autologous pseudoviruses							
animal sera	54A	6A5	DBO	OE5	DBO	O5E	
Pseudovirus	optC		ZM197M		CAP45		
Timepoint^2^							
t0	<10	<10	<10	<10	<10	<10	
t1	212	54	26	14	46	<10	
before t2	30	24	14	14	22	32	
t2	274	30	44	89	174	490	
*MLV control*	<10	14	16	11			
**Sera from 54A against heterologous pseudoviruses**							
	**Bal.26/** **B** ^**3**^	**ZM197M/** **C**	***CE1176/*** ***C***	***25710/*** ***B***	***X1632/*** ***G***	***X2278/*** ***B***	***398_F1_F6/*** ***A***
**timepoint:**							
t0	<10	<10	<10	<10	<10	<10	<10
t1	74	20	14	24	12	31	21
bef.t2	13	12	16	<10	14	>10	10
t2	100	28	61	63	100	93	91

^1^serum dilution resulting in 50% neutralization;

^2^immunization timepoint according to Fig. 2;

^3^HIV-1 subtype is underlined, Tier 2 isolates are in italic.

### Selection of nanobodies with broad neutralizing activity against heterologous Tier 2 HIV-1 subtypes from VHH phage libraries

VHH phage libraries were generated from all four animals starting from lymphocyte RNA from early and late immunization timepoints (t1 and t2, Fig. 2), respectively. After PCR amplification of VHH sequences from cDNA, cloning and plating, 95–100% of bacterial colonies were positive by colony PCR. Titers of the bacterial libraries ranged from 4 × 10^8^–2 × 10^9^ cfu/ml and the titers of the corresponding phage libraries after superinfection with Hyperphage were 1 × 10^12^ pfu/ml. After three rounds of selection on the autologous gp140 SOSIPs, about 1300 randomly picked clones were first analyzed by phage ELISA followed by soluble VHH ELISA. Eighty clones were found to bind in both settings. Sequencing of the VHH inserts revealed nanobodies from 25 different families.

For a rapid initial assessment of the neutralizing activity of the selected VHHs, periplasmic lysates of the 25 families were analyzed *in vitro* in the TZM-bl assay for neutralization of the pseudoviruses Bal.26 (subtype B, tier 1B) and optC (ancestral subtype C used as immunogen, Tier 2 (not yet determined officially)). Eight VHH clones showing neutralizing activity against one or both pseudoviruses were recloned into the pCAD51 expression vector (outside the PIII phage context), expressed in *E. coli* and purified via Ni-NTA chromatography. All eight sequenced VHHs show characteristic long CDR3 sequences^28^ in the range of 19 to 21 amino acids (Table 2) as well as the presence of a cysteine (except VHH-43) able to stabilize the extended CDR3 loop by pairing with a cysteine in CDR1.

**Table 2:** CDR3 sequences of selected nanobodies.

Name	CDR3 sequence	length (aa)	library (Fig. 2)
**VHH-1**	A D K W G G Y C G G V A D I A H F G Y	(19)	54A1
**VHH-5**	S T R V W G G Y C G G L D D A T N N D	(19)	54A1/DBO2
**VHH-A6**	T E T W G P Y C G: Q A S P A Y F P Y	(18)	DBO2
**VHH-9**	D V Y R G G Y C S A Y A G R:: F S Y	(17)	54A2
**VHH-28**	D V N R G G F C Y I E D W Y:: F S Y	(17)	54A2
**VHH-33**	D D L R G G Y C S S S P T L:: Y T Y	(17)	54A1
**VHH-23**	G T R P S I F C Y K G D R W:: Y S A	(17)	54A2
**VHH-43**	S Y Q F F S S E P S L S A R R: Y T D	(18)	54A1

Sequences were aligned by eye. The conserved cysteine forming a disulfide with a cysteine in CDR1 is underlined; “:” indicates deleted amino acids.

**Figure 4:**
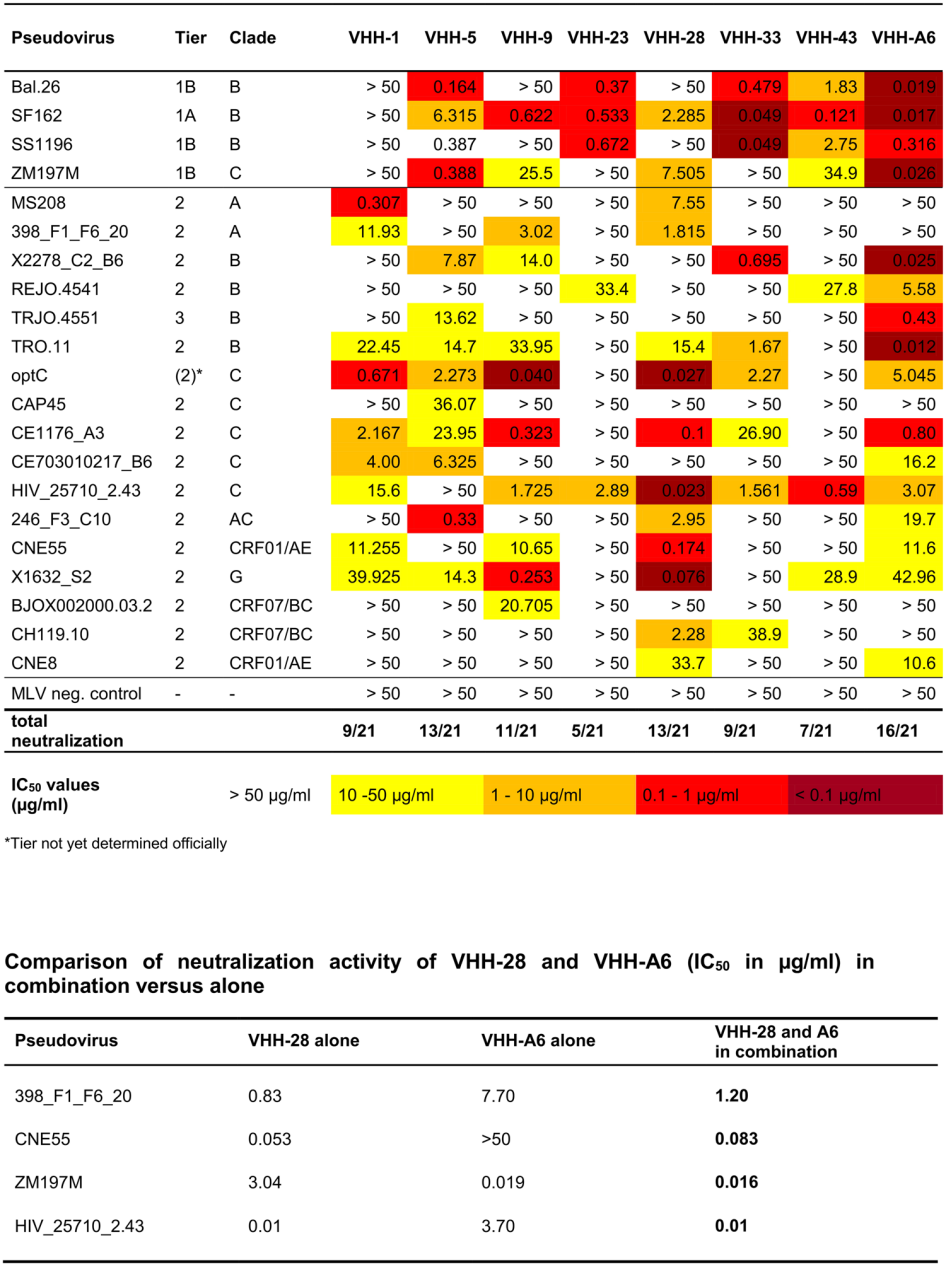
Neutralization activity of purified VHHs against a standard panel of pseudoviruses comprising different clades and neutralization sensitivities. VHHs were tested against a panel of pseudoviruses in the TZM-bl assay. Neutralization sensitivities are colour-coded according to the IC_50_ values: dark red corresponds to IC_50_ < 0.1 µg/ml, light red is from 0.1–1 µg/ml, orange is from 1–10 µg/ml and yellow is from 10–50 µg/ml. IC_50_ values were determined from the respective neutralization curves using GraphPad Prism and correspond to the mean IC50 values of 2–3 independent experiments. MLV pseudovirus was included as negative control. *Tier not yet determined officially. VHH28 and A6 were additionally tested in combination due to their complementary neutralization pattern.

The purified VHHs were then analyzed with respect to their neutralizing capacity against a panel of 21 pseudoviruses derived from several HIV-1 subtypes and neutralization sensitivities (4 tier 1, 16 tier 2 and 1 tier 3). Among these are all 12 strains from the extended global panel of HIV-1 reference strains for standardized assessment of vaccine-elicited neutralizing antibodies recently defined by deCamp *et al*.^45^. All eight VHHs showed neutralization of the more neutralization-resistant tier 2 pseudoviruses from at least two and up to six different subtypes including circulating recombinant forms (CRF) (Fig. 4). Interestingly, some VHHs showed complementary neutralization patterns: VHH-A6 with the broadest spectrum of neutralization capacity (16/21 including the tier 3 strain TRJO.4551) neutralizes most efficiently subtype B pseudoviruses, whereas VHH-28 neutralizes most efficiently subtype C, but also the strains from subtypes A and G. Thus, the complementary neutralization pattern of VHH-A6 and VHH-28 covers 19 strains from our virus panel of 21, including the epidemiologically most relevant subtypes C and A. To proof this complementary neutralization pattern, we exemplarily combined nanobodies VHH-A6 and VHH-28 in neutralization assays with 4 HIV-1 pseudoviruses differentially neutralized by the single VHHs. In all cases, the combination broadened the neutralization activity, as it neutralized each virus tested according to the more active nanobody in the combination (Fig. 4).

Surprisingly, VHH-1 did not neutralize the neutralization-sensitive tier 1 strains (3 subtype B and 1 subtype C), although it neutralized some tier 2 strains from subtypes A and C quite efficiently. Similarly, VHH-9 and VHH-28, the latter strongly neutralizing subtype C tier 2 strains, also neutralized only 1 of the 3 tier 1 subtype B strains and only weakly the subtype C tier 1B strain (ZM197M). Thus, nanobodies VHH-1, VHH-9 and VHH-28 rather seem not to recognize subtype B efficiently, irrespective of neutralization sensitivity.

For two of the broadest neutralizing VHHs, VHH-9 and VHH-28, binding to optC SOSIPs by ELISA (Supplementary Figure S5) correlated with potent neutralizing activity of the optC pseudovirus (IC_50_ 0.03–0.04 µg/ml) (Fig. 4). Interestingly, both nanobodies are derived from the phage library 54A2 at timepoint t2 after the late second immunization boost with optC SOSIP (Fig. 2). VHH-23, also derived from the same library, binds more weakly to optC SOSIP and shows a very narrow neutralization breadth: it does not neutralize the optC pseudovirus, but only the subtype B tier 1 viruses, one subtype C tier 2 strain and one subtype B tier 2 weakly (Fig. 4). VHH-A6 is also derived from a library of the late immunization timepoint (t2), but from animal DBO, which was immunized with the mixture of ZM197M and CAP45 SOSIPs. This nanobody shows intermediate binding to optC SOSIP, best binding to ZM197M (Supplementary Figure S5) and reveals the best neutralization breadth (16/21 strains including the tier 3 TRJO.4551). It also neutralizes the optC pseudovirus and the autologous ZM197M, but not CAP45. VHH-23 and VHH-43 bind optC SOSIP, but do not neutralize optC and show very limited neutralization breadth. In contrast, VHH-33, VHH-1 and VHH-5 show lowest binding to optC SOSIP, but neutralize optC pseudovirus with an IC_50_ of 0.7–3.2 µg/ml and display intermediate neutralization breadth. This may indicate a slightly better exposure of the epitopes in the context of the pseudovirus compared to the respective SOSIPs. The binding to optC SOSIP and the neutralization breadth of the VHHs with respect to the pseudovirus panel was similar when reformatted into the human IgG1 Fc antibody format (Supplementary Figure S6 and Supplementary Table S1).

### Determination of epitopes for broadly neutralizing nanobodies

**Figure 5:**
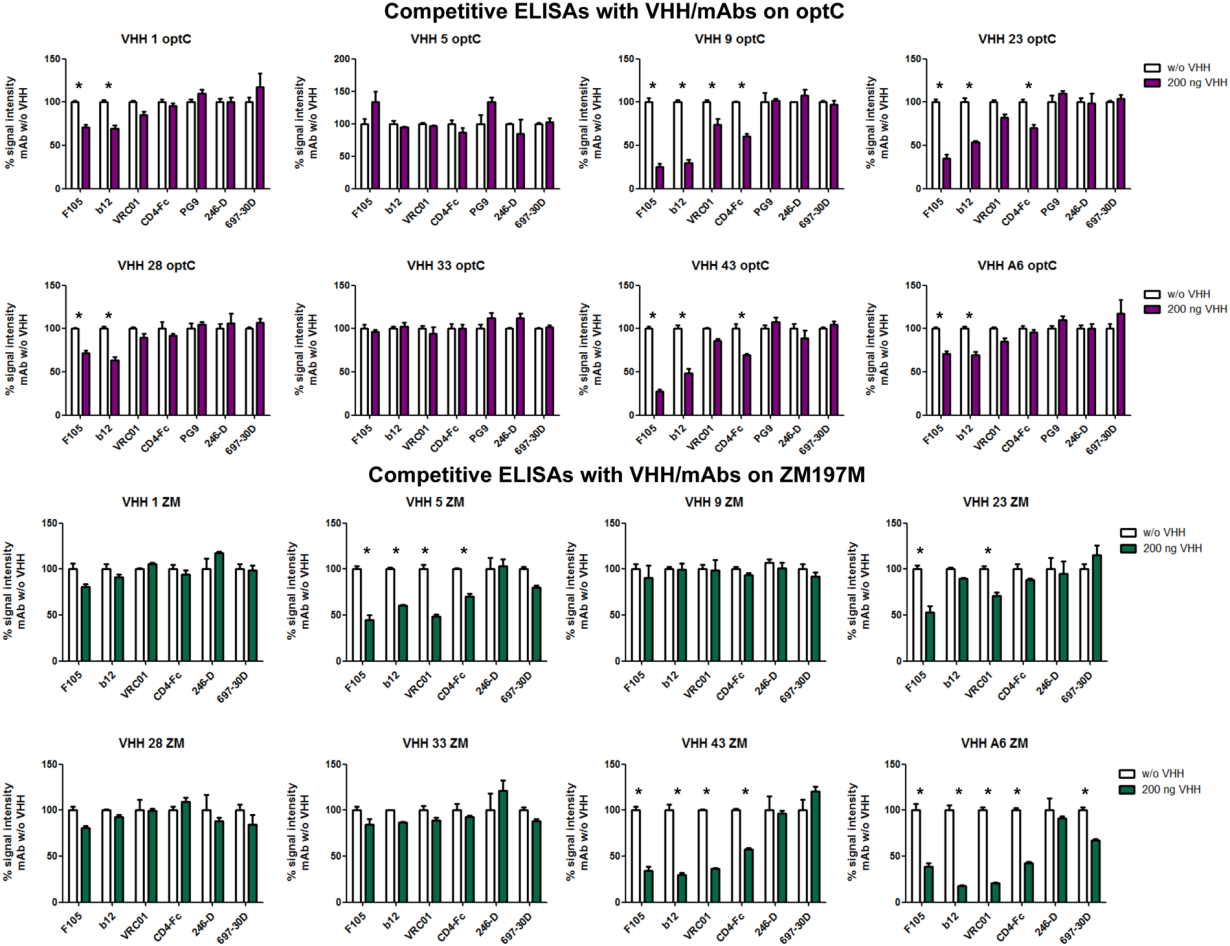
Competitive ELISA with VHH and known mAbs on optC and ZM197M SOSIPs. On optC (upper part), CD4bs ligands compete with VHH-9, VHH-23, VHH-43, A6, less with VHH-28 and VHH-1; on ZM197M (lower part), CD4bs ligands compete with A6, VHH-43, VHH-5, VHH-23, less/not with VHH-1, VHH-28, VHH-33. Detection of competition also depends on strength of VHH binding to the respective SOSIP (see Supplementary Figure S5: VHH-9 does not bind ZM197M −> no competition detectable). Stars indicate competition, see also legend to Supplementary Table S2.

The different neutralization patterns observed for the selected VHHs may be due to the recognition of different epitopes. We attempted to elucidate the epitopes targeted by the best neutralizing nanobodies first by competition ELISAs. These were performed with several monoclonal antibodies (mAbs) targeting well-known epitopes in Env including the CD4 binding site (CD4bs) (F105, b12, VRC01 CD4-Fc), CD4-induced (CD4i) epitopes (17b), the V2 apex (697–30D, PG9) or gp41 cluster I peptide (246-D), which we previously analyzed for binding to optC SOSIP (Supplementary Figure S1). Interestingly, except VHH-33, which did not compete with any of these mAbs, all the other VHHs competed with CD4bs ligands, although with different patterns (Fig. 5, Supplementary Table S2). VHH-33 does not bind to ZM197M SOSIP (Supplementary Figure S5); however, the fact that it binds and neutralizes optC suggests that this VHH recognizes an epitope different from those of the mAbs used for competition. In contrast, VHH-43 binds to both SOSIPs with intermediate strength, competes with most CD4bs mAbs on both, but only neutralizes mostly Tier 1 strains and HIV_25710_2.43 at low IC_50_. A similar pattern is seen for VHH-23; however, it binds only very poorly to ZM197M SOSIP consistent with a lack of neutralization of the ZM197M pseudovirus. For the other VHHs, the differential pattern of competition seen on optC and ZM197M SOSIPs with the CD4bs mAbs correlated quite well with differential binding of the VHHs to both SOSIPs: generally, VHHs bound better to optC SOSIP in agreement with their origin (library of animal 54A immunized with optC SOSIP) and the antigen they were selected with (optC) (Supplementary Figure S5). Only VHH-A6 originated from animal DBO immunized with ZM197M and CAP45 SOSIPs, which also served for selection. Nevertheless, VHH-A6 also showed good binding to optC SOSIP and overall broadest neutralization activity. VHH-A6 additionally competed with mAb 697–30D targeting V2^47^. VHH-9 and VHH-28 bound very well to optC SOSIP, but only weakly to ZM197M, which would explain why there is competition with CD4bs mAbs on the first, but not on the latter. VHH-1 behaved similarly; however, binding to optC SOSIP was much lower than for VHH-9 and VHH-28, as also reflected by reduced neutralization potential for optC pseudovirus. VHH-5 showed lowest binding to optC SOSIP and intermediate binding to ZM197M SOSIP and consequently only showed competition with CD4 binders on ZM197M SOSIP. Differences in the competition patterns of the VHHs with different CD4bs ligands on a given SOSIP may result from differences in the binding mode of the VHHs to the CD4 binding site.

**Figure 6:**
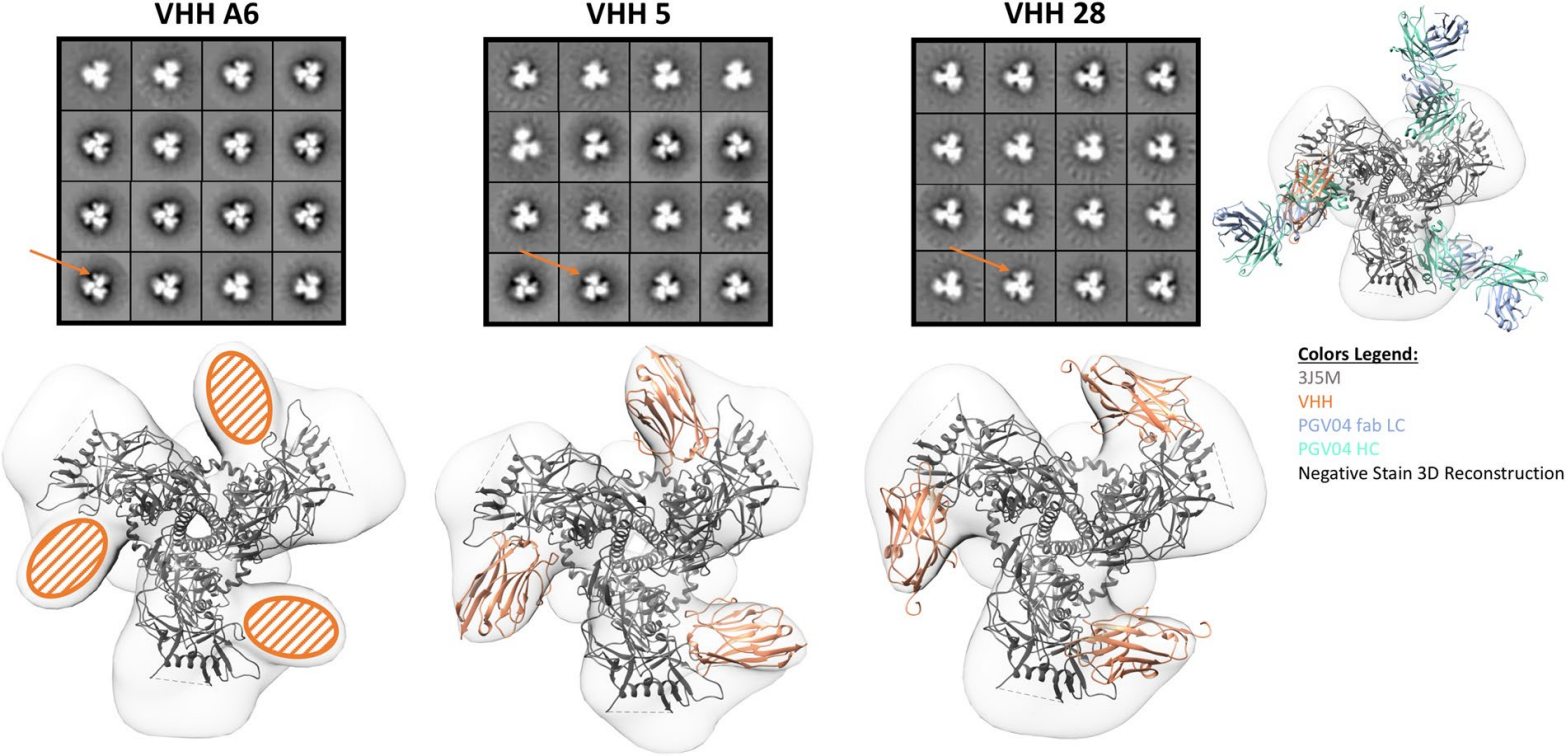
Negative stain electron microscopy reveals VHH’s target the CD4 binding site. Class averages from negative stain electron microscopy of ZM197M SOSIP trimers (version 5.2 donated by Rogier Sanders) complexed with the indicated VHH’s (upper row). In each complex three VHH’s (orange arrow) bind per trimer. A high resolution model of BG505 SOSIP.664-PGV04 Fab complex (PDB 3J5M) is docked into the 3D reconstructions of ZM197M SOSIP-VHH complexes (bottom row). Crystal structures of the VHH’s are in orange (A6 not yet available). The CD4 binding site Fab PGV04 is shown for comparison with the heavy chain in green and the light chain in blue. Alhough the VHH’s bind at a slightly different angle than PGV04, these data are consistent with the VHH’s targeting the CD4bs of the trimer.

Single particle negative stain electron microscopy (EM) was used to further characterize the epitopes of the VHHs. Consistent with the ELISA competition data, density was observed in the 2-dimensional class averages and 3-dimensional (3D) reconstructions corresponding to the VHHs proximal to the CD4 binding site (CD4bs) on the trimers (Fig. 6). 3D reconstructions of ZM197M-VHH A6, 5, and 28, each resolved at ~25 Å (Supplementary Figure S7), were used to dock in a high resolution model of BG505 SOSIP.664-PGV04 complex, which confirmed the presence of extra density at the CD4bs. Because of the small size of the VHHs and the low resolution of the EM 3D reconstructions, crystal structures of VHH-5 and 28 could not be independently docked into their respective EM density. However, comparison with the known CD4bs targeting bnAb PGV04, indicated that the VHHs bind at a slightly different angle than PGV04 Fab (Fig. 6).

### Crystal structures of VHH5 and VHH28

**Figure 7:**
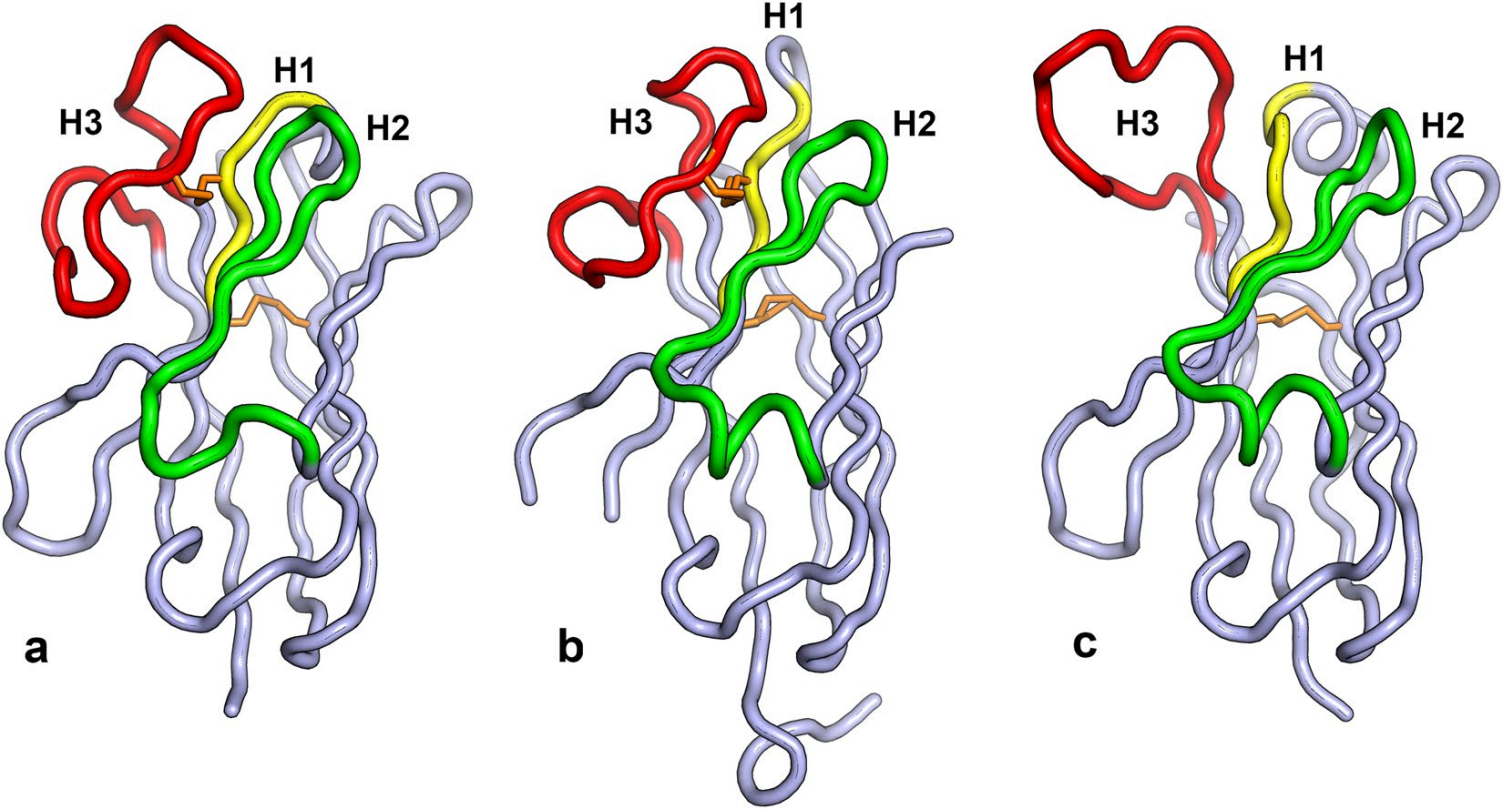
Crystal structures for VHH-5 and VHH-28. (**a**) The Cα trace for VHH-5 is shown with CDR’s labeled and colored yellow (H1), green (H2) and red (H3). This coloring scheme is used throughout the figure. The disulfide bonds linking CDR3 to CDR1 and residues H22 and H92 is colored in orange. (**b**) The Cα trace for VHH-28. Extra residues at the C-terminus belong to the 6-Histidine tag. (**c**) The Cα trace of the VH domain of human broadly neutralizing anti-HIV antibody PGV04, that also binds to the CD4 binding site.

Crystal structures were determined so far for VHH-5 (2.3 Å resolution) and VHH-28 (1.15 Å resolution, see Fig. 7); for A6 no crystal structure is available yet. Both, VHH-5 and VHH-28, have a long CDR3 region that folds over to partially mask the face of the VH domain that would be involved in contacts with the VL domain in a typical Fab fragment. The CDR3 of VHH-28 (19 residues long; H93-H102) is two amino acids shorter than that of VHH-5 (21 residues). In both structures, CDR3 is tethered to the body of the VHH by a disulfide bond between residue H33, and H100C or H100B, in VHH-5 or VHH-28, respectively. Although the same length (10 residues; H26-H35), the CDR1 loops in VHH-5 and VHH-28 adopt very different conformations, with VHH-5 CDR1 adopting the mouse/human canonical structure H1-10A^48^, while the VHH-28 CDR1 does not adopt any canonical structure, which is a common occurrence in camel VHHs^49^. CDR2 (H50-H58) is 9 residues long in VHH-5, and 10 residues long in VHH-28; however, the H52A insertion in VHH-28 is accommodated by a bulge in the loop around residues H60-H62, so that the CDR2’s are the same length at their tip, and both adopt the canonical structure H2–9A, with residues 52–55 forming a type-I β-turn in VHH-5, and 52, 52A, 53, 54 forming the same turn in VHH-28. Figure 7 shows the Cα traces of each VHH in comparison with the VH domain of Fab PGV04, a human broadly neutralizing antibody that targets the CD4 binding site.

## Discussion

In recent years, extremely potent antibodies with broad neutralizing potential across different clades of HIV-1 have been identified from a subset of chronically infected patients after several years of infection (summarized in the bNAber database^50^). These bnAbs have demonstrated protective immunity in a number of animal studies^20–23, 51–58^. Furthermore, if administered therapeutically in clinical trials, they recently were shown to reduce viremia^24, 59^ and to boost host immunity against HIV-1^25, 26^. Based on these results bnAbs themselves have entered clinical development for therapeutic and prophylactic applications. However, several grams of antibody are needed per injection and production of such large amounts of antibodies for clinical applications is tedious and cost-intensive. In this regard, VHH have several advantages including small size and their hydrophilic character allowing high yield production and good solubility^28–30^. Furthermore, the loop-structures of the three CDRs, often stabilized by a disulfide bond between CDR1 and CDR3, build a convex surface generating new types of paratopes compared to classical antibodies^60^; finally, VHH sequences belong to a single gene family with high homology to the human VH3 gene family.

Based on these favorable features of nanobodies, we aimed at selecting broadly neutralizing nanobodies against HIV-1 from dromedaries immunized with soluble trimeric gp140 SOSIP immunogens^2^. Such gp140 SOSIP trimers have previously been shown to mimic the native Env spike in numerous immunogenicity and structural studies^4, 5, 61^. During the course of our study, the group of Robin Weiss published the selection of broadly neutralizing nanobodies from llamas proving that immunization of camelids with trimeric gp140 can induce bn nanobodies against HIV-1^39–41^. Nanobodies with much less potency and breadth were selected by Lutje Hulsik *et al*. using proteoliposomes containing the membrane-proximal external region (MPER) of gp41 as immunogens in llamas^62^.

In this study, we derived gp140 SOSIPs from three HIV-1 subtype C Env sequences, including two recently transmitted strains and one reconstructed ancestral subtype C consensus sequence^43^. These strains are epidemiologically most relevant, as subtype C is predominating in the epidemic worldwide and immunogens derived from Env of recently transmitted strains should mimic antigens from those HIV strains whose transmission should be primarily prevented by a vaccine. The inclusion of the ancestral subtype C consensus sequence derived immunogen (optC) was intended to potentially broaden the antibody immune response due to conserved antigenic features. When comparing the two animals receiving the ADVAX adjuvant, 54A immunized with optC SOSIP and DBO immunized with the mixture of ZM197M and CAP45 SOSIPs, optC induced the strongest immune response in sera in terms of both, binding to the autologous immunogens (Fig. 3) and neutralization of autologous pseudoviruses (Supplementary Figure S3, Table 1). Interestingly, sera from timepoints t1 and t2 from animal 54A also neutralized moderately heterologous tier 2 pseudoviruses from the same subtype (C), but also from heterologous subtypes A, B and G (Table 1, Supplementary Figure S4). In contrast, t2 serum from animal DBO, which strongly neutralized the autologous subtype C pseudoviruses (Table 1), only neutralized the heterologous tier 1 Bal.26 subtype B strain (Supplementary Figure S4). As sera from animals 6A5 and O5E receiving the GERBU adjuvant during the first immunization cycle did only neutralize the autologous pseudoviruses, we concentrated on screening the VHH-libraries generated from the B-cells of animals 54A and DBO on the autologous SOSIP constructs.

Sequencing of 80 VHH clones binding to the autologous SOSIPs allocated the nanobodies to 25 different sequence families defined by 80–100% homology at the amino acid level. Nguyen *et al*. described 33 different VHH germline sequences to be present in a dromedary, indicating that we were able to select nanobodies across diverse sequence families in our screenings^60^. Among the 8 neutralizing nanobodies VHH-9, VHH-28 and VHH-A6 were most potent, neutralizing 11, 13 and 16 pseudoviruses of 6–8 different subtypes/CRFs from the 21 strains in the panel analyzed. VHH-A6 was not only the broadest, but also the most potent nanobody: besides neutralizing all 4 Tier 1 strains, it neutralized all subtype B strains (3 Tier 2, 1 Tier 3) in the range of 0.012–5.58 µg/ml, 4 out of 5 subtype C strains (Tier 2) with 3.07–16.2 µg/ml, 3 AC/AE recombinants (Tier 2) with 10.6–11.7 µg/ml and subtype G with borderline activity (42.96 µg/ml). Evidently, the neutralization patterns of VHH-28 and VHH-A6 show some degree of complementarity, as they cover all but two HIV-1 strains in the panel, including the epidemiologically most relevant subtypes C, A and B (Fig. 4). The only exceptions were CAP45 (subtype C) and BJOX002000.03.2 (subtype BC), which are difficult to neutralize, as only one of the 8 nanobodies shows neutralization with each of these strains with an IC_50_ above 20 µg/ml. The complementary neutralization pattern of the VHH-A6 and VHH-28 in combination could be proven experimentally in neutralization assays, in which both nanobodies were used alone and in combination and assessed for neutralization of 4 pseudoviruses of the panel differentially neutralized by the two single VHHs. The neutralizing activity of the combinations always was similar to the more active nanobody in the combination (Fig. 4), suggesting that this particular combination will indeed neutralize all isolates neutralized by either one of the single nanobodies according to Fig. 4, i.e. 19/21 (except CAP45 and BJOX002000.03.2, which are not neutralized by any of the two VHHs).

Nanobody VHH-5 with intermediate breadth and potency was selected from library 54A-1, i.e. from the early library of the dromedary immunized with optC SOSIP. Interestingly we could select a VHH (3.1) with 96% amino acid sequence identity also from the late library DBO-2 derived from the dromedary immunized with the mixture of ZM197M and CAP45 SOSIPs. This finding indicates that, despite the mechanisms for the generation of antibody diversity^60^, very similar nanobodies can be generated in different hosts upon immunization with similar, but different immunogens (Fig. 2).

When analyzing the selected VHHs for structural features such as length of CDR regions (Supplementary Figure S8), there was no significant difference in loop lengths between CDR1, CDR2 and CDR3 regions between neutralizing and non-neutralizing VHHs compared to random VHH sequences, although there may be a small tendency towards longer CDR3 loops in the neutralizing VHH. However, the CDR3 length (19.75 aa in the neutralizing 18.95 aa in the non-neutralizing group) is considerably longer than average HCDR3 length in human antibodies (12–13 aa); however, long HCDR3 lengths are also more frequently found in human Abs broadly neutralizing HIV-1^35^. 21 of the 25 VHHs have a cysteine in CDR1 and CDR3, respectively, to stabilize the paratope structure, whereas the other 4 VHHs lacked both cysteines. Interestingly, only one of these four, VHH-43, was in the neutralizing group, which together with VHH-23 is among the weakest neutralizers concerning breadth as well as potency (Fig. 4). Thus, stabilization of the paratope structure may be of particular importance for optimal binding and consequently neutralization.

Interestingly, the nanobodies linked to the CH2-CH3 Fc domain of human IgG1 resulted in comparable breadth of neutralization against the HIV-1 pseudovirus panel in the TZM-bl assay (Supplementary Table S1) indicating that reconstitution of a heavy chain formate by linkage of the VHH to an Fc domain does not interfere with neutralization. Although this may not be too surprising, since the paratope did not change, it emphasizes that Fc-mediated effector functions can potentially be linked to these nanobodies besides their neutralizing activity, if necessary in view of clinical applications.

Binding competition experiments with mAbs with well known epitopes showed that, except VHH-33, all VHHs competed with CD4bs mAbs and CD4-Fc, although to a different degree (Fig. 5, Supplementary Table S2). This is surprising, as in contrast to previous screenings for HIV-specific VHHs^40, 41^, our screening was not specifically designed to favor the selection of CD4bs VHHs by competitive elution; it therefore seems likely that the CD4 binding site may be preferentially targeted by nanobodies, probably due to their small size and/or to the intrinsic properties mimicking the contact residues of conventional human HIV neutralizing mAbs targeting the CD4bs, mostly via the heavy chain region^16^. It may, however, also be due to the fact that both studies used SOSIP constructs from the first generation, i.e. including most of the MPER of gp41; due to the hydrophobicity of this region these SOSIPs tend to aggregate, which influences the structure of the variable domains in the trimer resulting in a slightly more open configuration, as also reflected by the binding behavior of neutralizing and non-neutralizing mAbs (Supplementary Figure S1). Indeed, the newer slightly shortened generation of SOSIPs (SOSIP.664) developed during the course of this study is recognized by a number of bnmAbs targeting conformation-sensitive epitopes in the variable domains^3, 5, 10^. It remains to be seen, if the newer SOSIP versions will select for VHHs targeting epitopes other than the CD4bs.

Studies to achieve crystals of additional VHHs and the VHH-Env complexes are ongoing to elucidate further structural details about the binding mode of the selected VHHs to their target epitopes in Env. The complementary and broad neutralization patterns of VHH-A6 and VHH-28 across 19 of 21 HIV-1 strains of different subtypes from our panel strongly suggests that these nanobodies are excellent candidates for further clinical development for prophylactic and therapeutic applications. In particular, vector-mediated local expression of combinations of secreted nanobodies at mucosal sites, where HIV-1 transmission occurs, would be an option to achieve prevention of HIV-1 infection in high risk cohorts.

## Methods

### Cloning and expression of recombinant HIV-1 SOSIP Env proteins

ZM197M, CAP45 and optC gp160 expressing plasmids were obtained through the AIDS Reagent Program, Division of AIDS, NIAID, NIH from Drs. B. H. Hahn, Y. Li and J. F. Salazar-Gonzalez: ZM197M.PB7, SVPC6; Drs. L. Morris, K. Mlisana and D. Montefiori.: CAP45.2.00.G3, SVPC16; pAncCgp160-opt (Cat#11399) from Dr. Beatrice Hahn^43^. The corresponding gp140 SOSIP constructs were generated via SOE-PCR to introduce amino acid substitutions at A501C, I559P and T605C (HXB2 numbering) as well as a stop codon upstream of the transmembrane domain^2^ followed by cloning into the pEE12.4 expression vector (Lonza Walkersville, USA). 70 µg *env* DNA and 35 µg furin DNA (Imagenes-bio, Germany) were used to transiently transfect 2 × 10^7^ 293T cells (ATCC® CRL-11268™) using 210 µl polyethylenimine (PEI, 1 mg/ml, Sigma-Aldrich). Supernatants were harvested after 48 hours and gp140 SOSIP proteins were purified via lectin (*Galanthus nivalis*, Sigma-Aldrich) affinity chromatography (elution with 10 ml 0.5 M methyl-α-D-mannopyranoside)^63^. Trimeric gp140 was further purified by size exclusion (HiLoad™ 16/60 Superdex™ 200, GE Healthcare) and anion exchange chromatography (HiTrap™ DEAE FF, GE Healthcare). The quality of purified SOSIPs was controlled after separation in SDS-gels followed by silver staining, reactivity with known mAbs and negative stain electron microscopy.

### Immunization of *Camelus dromedarius*, PBMC isolation and cDNA preparation

Four dromedaries (designated 54A, 6A5, DBO and O5E) were immunized at the Central Veterinary Research Laboratory (CVRL, Dubai, UAE) with gp140 SOSIP Env proteins. All experimental animals and their treatment in this study were reviewed and approved by the Animal Ethic Committee. The welfare of all experimental animals and treatment of them conducted by the Central Veterinary Research Laboratory (CVRL) were reviewed and approved by the Animal Ethic Committee of CVRL and Ministry of Climate Change and Environment (MOCCAE) of the United Arab Emirates (Permit Number: 550353). Animals were immunized subcutaneously once a week for seven weeks (1st immunization cycle) and boosted once seven months later (2^nd^ immunization t2). Dromedaries 54A and 6A5 received boosts of 100 µg optC SOSIP, whereas DBO and O5E received a mixture of 50 µg ZM197M and 50 µg CAP45 SOSIPs. As adjuvants, Advax™ (Vaxine Pty Ltd, Australia) was used for 54A and DBO and GERBU FAMA (GERBU Biotechnik GmbH) for 6A5 and O5E during the first immunization period. All four dromedaries received Advax™ for the second immunization. Serum samples were taken after every protein boost to monitor the immune response by ELISA. Blood samples (25 ml) drawn after the 1^st^ immunization cycle and the 2^nd^ immunization were used to isolate PBMCs via Pancoll® gradient centrifugation. Aliquots of 1 × 10^7^ cells were used to extract RNA using the RNeasy Mini Kit (Qiagen) according to manufacturer’s instructions. cDNA was synthesized from 8 µl (about 3 µg) RNA using SuperScriptIII (Life Technologies GmbH) and random hexamer primers.

### VHH phage library construction

VHH genes were amplified from dromedary cDNA via a nested PCR using primers cVHH_Lead_for (GTCCTGGCTGCTGCTCTTCTACAAGG), cVHH_CH2_rev (GGTACGT GCTGTTGAACTGTTCC), cVHH_FR1_for (TCGCGGCCCAGCCGGCCATGGCAGATGTGC AGCTGCAGGAGTCTGGRGGAGG) and cVHH_FR4_rev (GGACTAGTGCGGCCG CTGAGGAGACGGTGACCTGGGT). After restriction with SfiI and NotI, the PCR inserts were ligated into the similarly digested phagemid vector pHEN2^64^ that incorporates the pIII gene and C-terminal 6xHis-tag and myc-tag. The ligation product was electroporated into TG1 cells (Source BioScience, UK). The corresponding phage library was produced by superinfection of the bacterial library with Hyperphage M13 K07ΔpIII (PROGEN Biotechnik GmbH). Phage were precipitated (20% PEG in 2.5 M NaCl) and titrated on TG1 bacteria.

### Biopanning and VHH selection

HIV-1 Env SOSIP specific VHHs were selected by phage display after immobilization on magnetic beads (Dynabeads® M-280 Tosylactivated, Invitrogen) or ELISA plates (Microlon, Greiner Bio-One). For selection on magnetic beads, 1 × 10^12^ phages were first subjected to negative selection on 2 × 10^7^ beads coated with supernatants from untransfected 293T cells (293Tneg) for 4 hours at room temperature. Afterwards, the phage containing supernatant was transferred to 2 × 10^7^ gp140 SOSIP coated (3 µg) beads and incubated overnight at 4 °C. Beads were washed 10 times with PBSTG and bound phages were eluted with 200 µl trypsin (10 µg/ml, Sigma-Aldrich) and used to infect TG1 cells. The resulting bacterial library was used to generate phage libraries by superinfection with M13KO7 helper phages (Invitrogen, Germany) at an MOI of 15 for the next round of biopanning. Three selection rounds were performed.

For selection on ELISA plates, 1 × 10^11^ phages were first subjected to negative selection in a well of a 96 well ELISA plate coated with 293Tneg for 4 hours at room temperature. Afterwards, the supernatant was transferred into a well coated with 3 µg gp140 SOSIP Env and incubated overnight at 4 °C. Wells were washed 15 times with 0.05% PBST. Elution and generation of the phage libraries for the next rounds of selection were performed as described above.

### Phage ELISA and soluble VHH ELISA

After selection, randomly picked clones were analyzed regarding their binding to gp140 SOSIP by ELISA. 96 well plates filled with 200 µl 2YT-Amp/2% glucose medium were inoculated with single clones per well or appropriate controls. Plates were incubated at 37 °C and 250 rpm overnight and used to inoculate new 96 well culture plates for monoclonal phage production. Plates were incubated 2 hours at 37 °C, 250 rpm after addition of 20 µl of the overnight culture. 10^9^ M13KO7 helper phages were added per well and infection of bacteria was carried out for one hour at 37 °C and 250 rpm. Plates were centrifuged at 5000 g for 10 min and cell pellets were resuspended in 200 µl 2YT-Amp/Kan per well. The plates were incubated at 30 °C, 250 rpm overnight. After centrifugation, 45 µl of the phage-containing supernatant was transferred to gp140 SOSIP coated (0.2 µg/well) and blocked (5% MPBS) ELISA plates for one hour. Between all steps, ELISA plates were washed three times with 0.05% PBST. Subsequently, anti-M13-HRP (GE Healthcare) was added for one hour to detect bound phages after addition of 50 µl Sure Blue TMB substrate (KPL Inc., USA) per well. The enzymatic reaction was stopped with 50 µl 0.5 M H_2_SO_4_. Absorption at 450 nm and 650 nm was determined with the ELISA Reader SpectraMax 340 (Molecular Devices Corp., USA).

Positive clones from phage ELISA were further analyzed outside the phage context by soluble VHH ELISA. 20 µl overnight culture were used to inoculate single wells of a 96 well plate filled with 180 µl 2YT-Amp/0.2% glucose medium and grown at 37 °C, 250 rpm. After 2 hours, 1 mM IPTG (final concentration) was added per well for induction of VHH expression and plates were further incubated for 4–6 hours followed by centrifugation. Supernatant was discarded and cell pellets were frozen at −20 °C for at least one hour. After thawing for 15 min, 100 µl PBS was added to the wells and periplasmic lysates containing the VHH-pIII fusion proteins were recovered by shaking at 700 rpm at room temperature. Plates were again centrifuged and 30 µl of the VHH-containing supernatant was transferred to gp140 SOSIP Env coated (0.2 µg/well) and blocked (5% MPBS) ELISA plates for one hour. Between all steps, ELISA plates were washed three times with 0.05% PBST. Bound VHHs were detected by addition of anti-c-myc antibody (AbD Serotec, USA) followed by a-mouse-HRP (GE Healthcare).

### Soluble VHH production

Positive VHH clones were subcloned into the pCAD51 expression vector (contributed by R. Weiss, University College, London) that incorporates a C-terminal 6xHis-tag and myc-tag and transformed into *E. coli* HB2151 cells (Source BioScience, UK). For crystallization studies we used a myc-tag deleted version. Overnight cultures were diluted in 250 ml LB-Amp/0.1% glucose medium, grown at 37 °C to an OD_600_ of 0.5–0.7 and induced with IPTG (final concentration: 0.5 mM). Bacterial cultures were incubated at 28 °C, 180 rpm overnight. Cell pellets were resuspended in 12 ml TES buffer (0.2 M Tris, 0.65 mM EDTA, 0.5 M sucrose, pH 8) and incubated on ice for one hour. Subsequently, 25 ml TES/4 buffer (TES buffer diluted 1:4 in dH_2_O) was added and incubated on ice for further 45 min. After centrifugation at 10,000 g for 30 min, the VHH-containing supernatants were applied to 1 ml HF Select Ni-NTA agarose columns (Sigma-Aldrich). His-tagged VHHs were purified from the periplasmic lysates according to manufacturer’s instructions. Eluted VHHs were concentrated and buffer exchanged to PBS using Amicon® Ultra-4 centrifugal filters (10 kDa cut-off; MerckMillipore). Protein concentration was determined via the Pierce™ BCA Protein Assay Kit (Thermo Fisher Scientific) according to manufacturer’s instructions.

### Cloning and expression of recombinant VHH-Fc proteins

To construct VHH-Fc fusion proteins, VHH sequences were recloned into the pCMX expression vector (contributed by M. Hust, TU Braunschweig), which incorporates an optimized human IgG1 Fc domain. 20 µg VHH-Fc plasmid DNA and 60 µl PEI (1 mg/ml) were used to transiently transfect 5 × 10^6^ 293T cells. Supernatant was harvested after 48 hours and once again after further 48 hours. Recombinant VHH-Fc proteins were purified via Pierce™ Protein A Agarose columns (Thermo Fisher Scientific) according to manufacturer’s instructions. Eluted VHH-Fc proteins were concentrated and buffer exchanged to PBS as described for VHHs.

### Immune response ELISA

Antibody response of immunized dromedaries was determined by ELISA. The respective gp140 SOSIP antigen (0.2 µg) was immobilized on ELISA plates overnight at 4 °C. The following steps were performed at room temperature with 3 washes (0.05% PBST) in between. Plates were blocked with 5% MPBS for one hour, followed by addition of dromedary sera at indicated dilutions. After one hour, Protein A-HRP (Sigma-Aldrich) diluted 1:25.000 was applied for detection with 50 µl Sure Blue TMB substrate (KPL Inc., USA) per well. The enzymatic reaction was stopped with 50 µl 0.5 M H_2_SO_4_. Absorption at 450 nm and 650 nm was determined (SpectraMax 340, Molecular Devices Corp., USA).

### VHH and VHH-Fc ELISA

0.2 µg of gp140 SOSIP Env was immobilized on ELISA plates overnight at 4 °C. The following steps were performed at room temperature with 3 washes (0.05% PBST) in between. Plates were blocked with 5% MPBS for one hour, followed by addition of VHH or VHH-Fc proteins at indicated concentrations. VHHs were detected via anti c-myc antibody (AbD Serotec, USA) and anti-mouse-HRP (GE Healthcare), whereas VHH-Fc proteins were detected via anti-human-Fc HRP (Jackson ImmunoResearch, UK).

### Competition ELISAs

To get first insight into the Env epitopes targeted by the VHHs, competition ELISAs were performed with known mAbs. 0.2 µg of gp140 SOSIP Env was immobilized on ELISA plates overnight at 4 °C. When CD4 inducible antibodies were used, gp140 SOSIP was preincubated with soluble CD4 (sCD4) (Sinobiologicals) (20 ng per 0.1 µg Env) for 30 min at 37 °C before coating. The following steps were performed at room temperature with 3 washes (0.05% PBST) in between. Plates were blocked with 5% MPBS for one hour, followed by addition of VHH proteins at indicated concentrations. After one hour, mAbs b12, VRC01, 17b, 246-D, F105 (AIDS Reagent Program, Division of AIDS, NIAID, USA), PG9 (POLYMUN Scientific, Austria) and CD4-Fc (Abcam, UK) were applied at a final concentration between 50 and 100 ng/well. HIV-1 mAbs were detected by addition of anti-human-HRP.

### Neutralization assays

Neutralization capacity of dromedary sera, VHHs and VHH-Fc proteins was determined via a standardized TZM-bl assay^46^. All HIV-1 Env pseudotyped viruses used in the experiments were obtained from the HIV Specimen Cryorepository (H. von Briesen, Fraunhofer IBMT, St. Ingbert)^45, 65^.

Dromedary sera were heat-inactivated at 56 °C for one hour prior to use. Three-fold serial dilutions starting with 1:10 were prepared from the dromedary sera derived from indicated time points. 50 µl pseudovirus (15,000 RLU) was added to the diluted sera and incubated at 37 °C for 60–90 min. 1 × 10^4^ TZM-bl cells were applied per well (containing 12.5 µg/ml DEAE-dextran) and grown for 48 hours. Cells were lysed in harvest buffer (2.5 ml glycerol, 1.25 ml 1 M MES-Tris pH 7.8, 25 µl 1 M DTT, 250 µl 10% Triton X-100, up to 25 ml dH_2_O) for 10 min at room temperature and frozen at −80 °C for at least one hour. Luminescence was measured by mixing 50 µl luciferin solution (25 µl luciferase buffer (6.25 ml 1 M MES-Tris, 1.25 ml 1 M MgCl_2_, 120 mg ATP, 42.5 ml dH_2_0) and 25 µl luciferin (10 mg luciferin, 36 ml 5 mM K_2_HPO_4_/KH_2_PO_4_, pH7.8)) with 60 µl thawed cells via a Lumistar Galaxy plate reader (BMG LABTECH GmbH). Neutralization was determined as the reduction in RLU in sample wells compared to RLU in virus control wells after subtraction of background luminescence. The 50% inhibitory dose (ID_50_) was defined as the reciprocal plasma dilution resulting in 50% reduction in RLU compared to virus control wells.

Purified VHH and VHH-Fc proteins were assayed in duplicates for neutralization in three-fold serial dilutions starting with a concentration of 50 µg/ml. IC_50_ values were determined using Prism software (GraphPad Software, Inc., San Diego, CA). For the nanobody combinations, both nanobodies were used at equal concentrations ranging from 10 µg/ml to 0.001 µg/ml for each nanobody. IC_50_ determinations were calculated according to the concentration of each single nanobody (i.e. an IC50 of 1 µg/ml of the combination corresponds to 1 µg/ml of each nanobody).

### Single Particle Negative Stain Electron Microscopy

Purified VHH’s were incubated overnight at room temperature with ZM197M SOSIP trimers (here the more advanced version 5.2 SOSIP kindly donated by Rogier Sanders, University of Amsterdam, was used) at a six-fold molar excess ratio. Complexes were deposited on glow-discharged carbon coated copper mesh grids, stained with 2% uranyl formate, and screened for the correct stain thickness and particle density with a FEI Morgagni (80 keV) electron microscope.

Data were collected on a FEI Tecnai Spirit T12 (120 keV) electron microscope with a Tietz TVIPS CMOS (4 K × 4 K) camera and the Leginon software^66^. Images were collected at −1.30 μm defocus at 52,000x magnification resulting in a pixel size of 2.05. The data were stored and processed within the Appion database^67^. Single particles were picked with the DogPicker software, stacked using a box size of 160 and binned by 2. Reference free 2D class averages were generated by the Iterative MSA/MRA software^68^. Single particles corresponding to complexes were selected for 3D refinement in EMAN2^69^, using a low pass filtered HIV trimer reconstruction as a starting model. Single particle counts for ZM197M SOSIP complexed with the VHH’s were: 21,751 for VHH28, 6,923 for VHH5, and 7,824 for VHHA6.

The figures associated with the electron microscopy data were generated with UCSF Chimera^70^.

### Crystal structure determination

VHH-5 and VHH-28 were both crystallized with an intact C-terminal 6-Histidine tag. VHH-5 was crystallized at 20 °C in a 96-well sitting drop plate, with precipitant 1.9 M ammonium sulfate, 0.1 M bicine, pH 9.0. VHH-28 was crystallized at 22 °C in a 24-well sitting drop plate (Cryschem Inc.), with precipitant 0.1 M sodium citrate, 0.1 M CHES, pH 9.5. Both crystals used for data collection were cryoprotected by brief immersion in the well solution augmented with 30% glycerol. Data for both crystals were collected at SSRL beamline 12–2 using a Pilatus 6 M detector. VHH-5 data were processed with XDS^71^, while the VHH-28 data were processed using HKL-2000^72^. The VHH-28 structure was determined by molecular replacement using Phaser^73^ with chain B of 4W6Y (Nanobody NbFedF9)^74^ as a search model. The structure was refined with Phenix^75^ to a resolution of 1.15 Å resolution using anisotropic B-values for all non-solvent atoms. The VHH-5 structure was phased in a similar manner using the VHH-28 coordinates as a model and refined to a resolution of 2.3 Å with Refmac5^76^ with isotropic B-values. All data collection and refinement statistics are included in Supplementary Table S3.

### Data availability

Structural data and coordinates are deposited in the PDB with accession codes 5U64 (VHH28) and 5U65 (VHH5) to be released upon publication.

Sequence data of the VHHs are deposited in GenBank with accession numbers KY363288 to KY363295 for VHH-1, VHH-5, VHH-9, VHH-23, VHH-28, VHH-33, VHH-43 and VHH-A6, respectively, to be released upon publication.

## Electronic supplementary material

Supplementary information
