# Supplementary material for: Selection of nanobodies with broad neutralizing potential against primary HIV-1 strains using soluble subtype C gp140 envelope trimers
Source: Sci Rep. 2017 Aug 21;7:8390. doi: 10.1038/s41598-017-08273-7 (PMC5566552; doi:10.1038/s41598-017-08273-7)
Supplement: Supplementary file 1 — Supplementary information [file 41598_2017_8273_MOESM1_ESM.pdf]

Supplementary Information

Selection of nanobodies with broad neutralizing potential against primary HIV-1 strains using soluble subtype C gp140 envelope trimers

Kathrin Koch, Sarah Kalusche, Jonathan L. Torres, Robyn L. Stanfield, Welbeck Danquah, Kamal Khazanehdari, Hagen von Briesen, Eric R. Geertsma, Ian A. Wilson, Ulrich Wernery, Friedrich Koch-Nolte, Andrew B. Ward, Ursula Dietrich

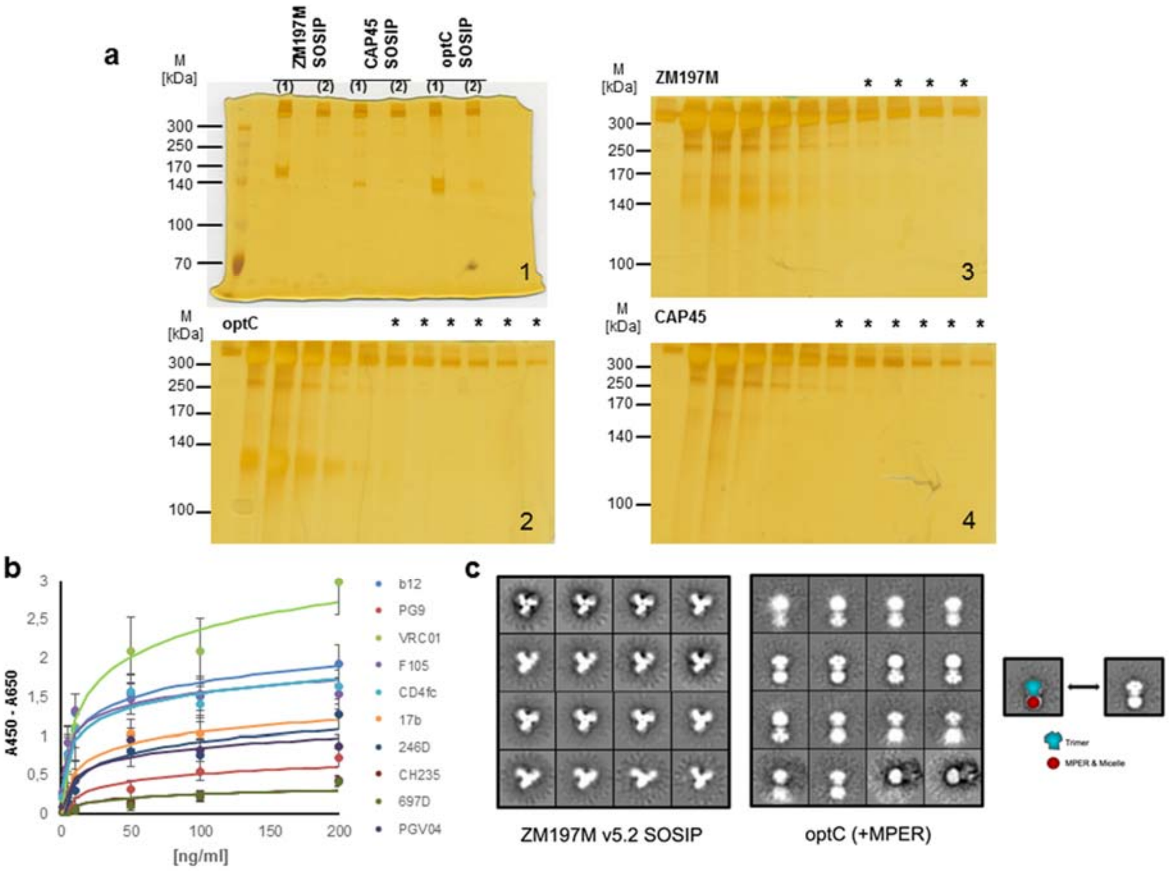

Supplementary Figure S1: Characterization of the SOSIP immunogens after size exclusion (SEC) and anion exchange chromatography (AEC). (a) Silver stainings of (1) the original gel shown in Fig. 1 showing the three SOSIPs after lectin affinity (lanes 1) and SEC and AEC (lanes 2) purifications; (2) –(4): Silver stainings of gels with the gradient fractions separated by SDS-PAGE. Fractions marked with an asterisk correspond to the trimer-enriched fractions used as immunogens in the dromedaries. Gels have been cropped below the 100 kDa marker and the marker lanes have been substituted by indicating the molecular weights. **(b)** Binding of mAbs/sCD4-Fc to optC SOSIP determined by ELISA. Detection was done by an anti-human Fc HRP-labeled mAb. Error bars represent SEM of triplicates. **(c)** Negative stain electron microscopy of the first generation optC SOSIP trimers (including the hydrophobic MPER, right) used as the immunogen and the more recent ZM197 v5.2 SOSIP (lacking the MPER, left) used in Fig. 5 for the VHH-SOSIP/ZM197M complexes. The optC SOSIP trimers (blue) that contain the hydrophobic gp41 MPER have micelles associated at the trimer base (red).

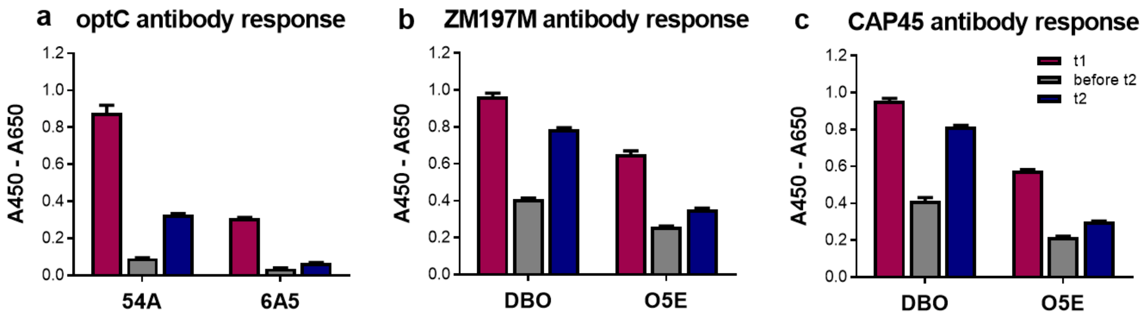

Supplementary Figure S2: Comparison of antibody responses in dromedaries immunized at early (t1) and late (t2) timepoints. ELISA experiments showing **(a)** optC antibody response in sera from 54A (diluted 1:1000) and 6A5 (1:500), **(b)** ZM197M antibody response and **(c)** CAP45 antibody response in sera from DBO (1:100) and O5E (1:250). t1 (purple): serum sample after the last immunization boost of the first seven immunizations; before t2 (gray): serum sample before the additional second protein boost; t2 (blue): serum sample after the additional second protein boost (see Fig. 2). One representative experiment of two independent experiments is shown. Error bars represent SEM from triplicates.

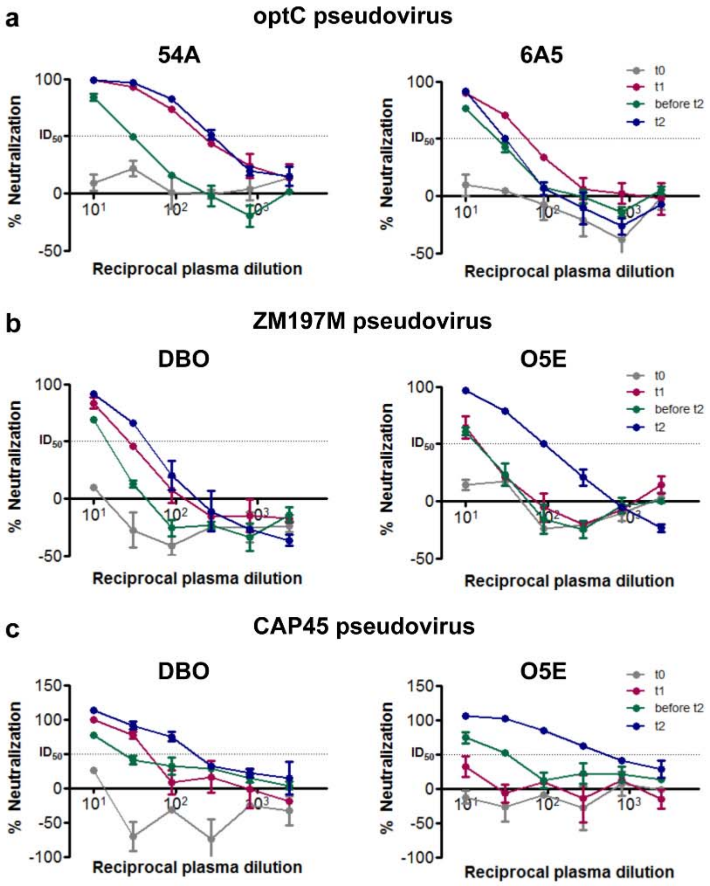

Supplementary Figure S3: Neutralization activity of dromedaries´ sera against autologous pseudoviruses. TZM-bl neutralization assays with sera from **(a)** 54A and 6A5 tested against the optC pseudovirus, **(b)** DBO and O5E tested against the ZM197M pseudovirus and **(c)** DBO and O5E tested against the CAP45 pseudovirus. Dotted lines indicate ID_{50} values. Starting dilution was 1:10. t0 (gray): preimmune serum; t1 (purple): serum sample after the last boost of the first immunization cycle; before t2 (green): serum sample before the second protein boost 7 months later; t2 (blue): serum sample after the second protein boost. One representative experiment out of two independent experiments is shown. Error bars represent SEM of duplicates.

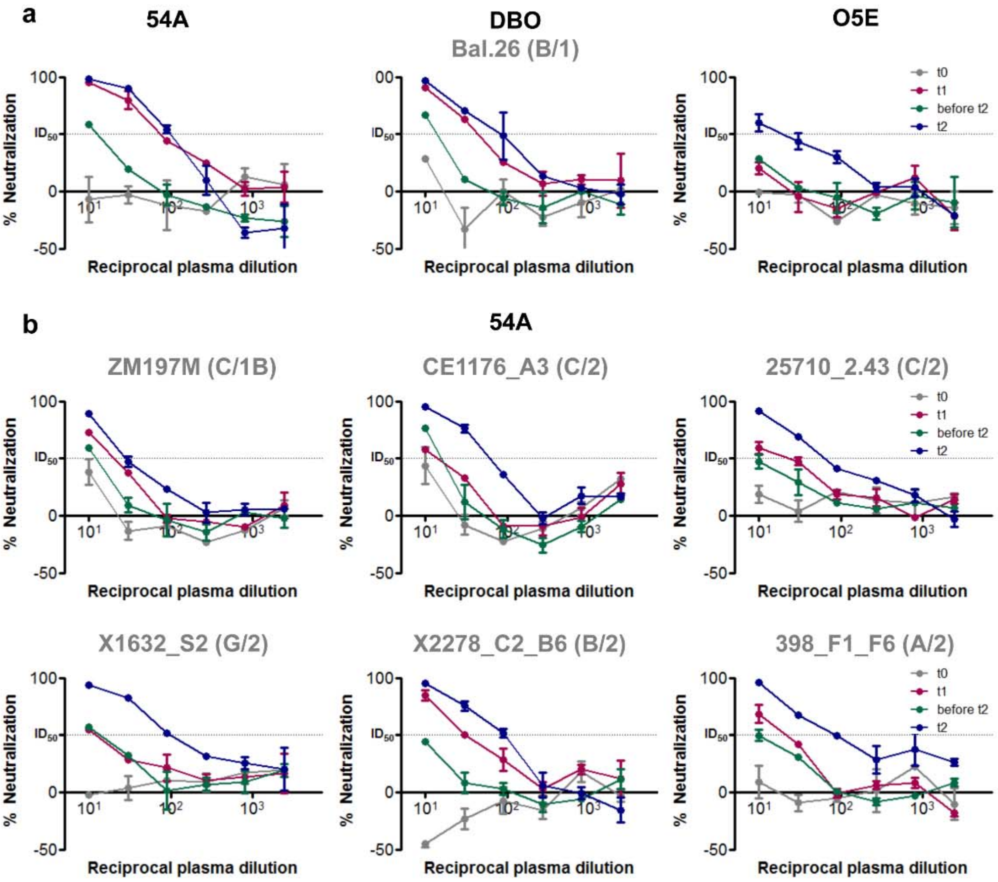

Supplementary Figure S4: Neutralization activity of dromedaries´ sera against heterologous pseudoviruses. TZM-bl neutralization assays with sera from **(a)** 54A, DBO and O5E tested against the Bal.26 pseudovirus (B/1), **(b)** 54A sera tested against pseudoviruses ZM197M (C/1), CE1176_A3 (C/2), 25710_2.43 (C/2), X1632_S2 (G/2), X2278_C2_B6 (B/2), 398_F1_F6 (A/2). Dotted lines indicate ID_{50} values. Starting dilution was 1:10. t0 (gray): preimmune serum; t1 (purple): serum sample after the last boost of the first immunization cycle; before t2 (green): serum sample before the second protein boost 7 months later; t2 (blue): serum sample after the second protein boost. For each pseudovirus (gray) the subtype and Tier is indicated in parentheses (i.e. 398_F1_F6 (A/2) means HIV-1 subtype A and Tier 2). One representative experiment out of two independent experiments is shown. Error bars represent SEM of duplicates.

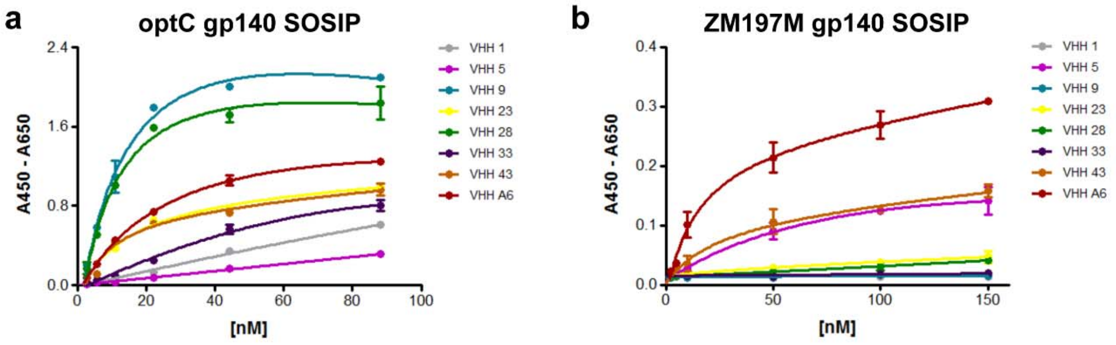

Supplementary Figure S5: Binding of selected VHHs to SOSIPs determined by ELISA. Binding of indicated VHHs to optC gp140 SOSIP **(a)** and ZM197M **(b)** was detected via the C-terminal myc-tag. Gray: VHH 1, pink: VHH 5, blue: VHH 9, yellow: VHH 23, green: VHH 28, purple: VHH 33, brown: VHH 43, pink: VHH A6. Error bars represent SEM of triplicates.

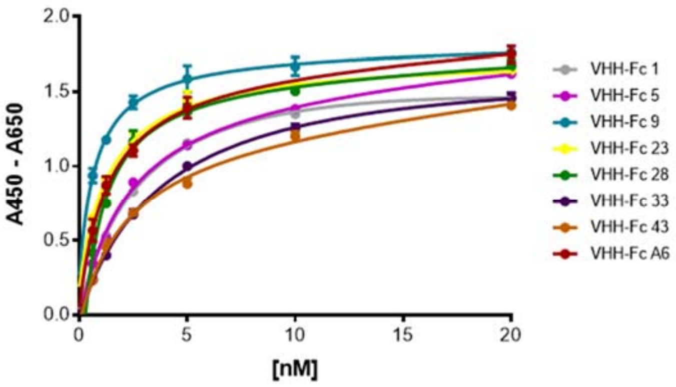

Supplementary Figure S6: Binding of VHH-Fc to optC SOSIP determined by ELISA. Binding of VHH-Fc proteins was detected by an anti-human Fc HRP-labeled mAb. Colours are as indicated besides the figures. Error bars represent SEM of triplicates.

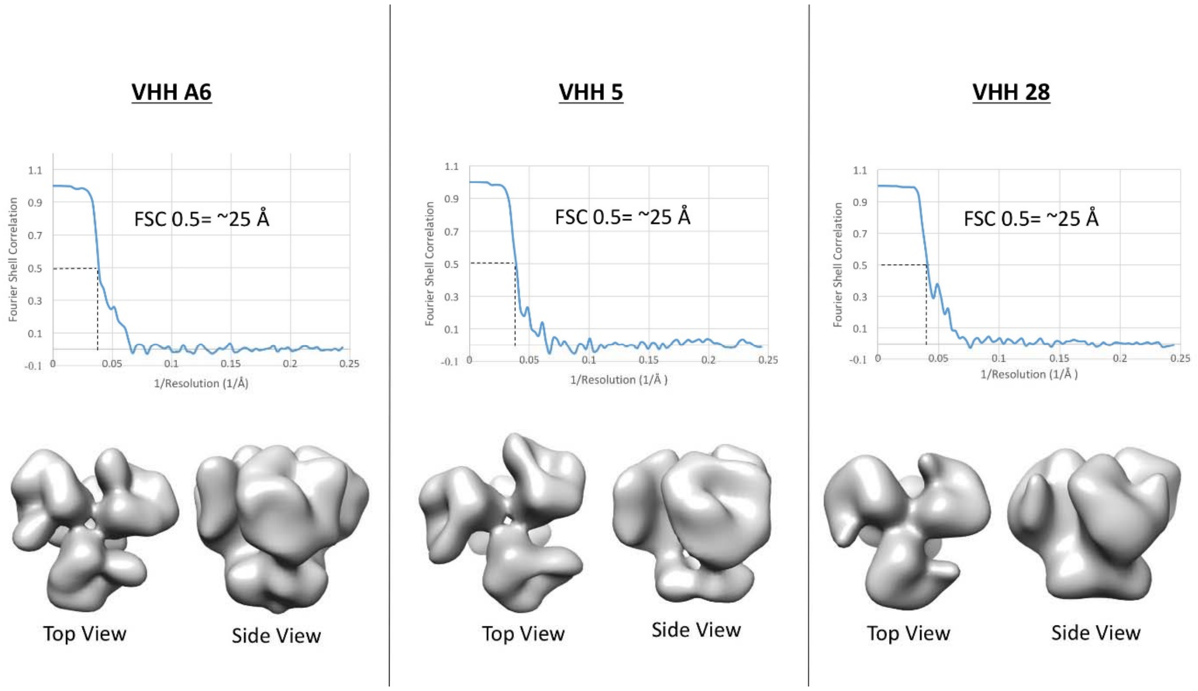

Supplementary figure S7: Fourier shell correlation curves of negative stain reconstructions of ZM197M-VHH A6, 5, and 28. Final calculated resolutions for each single particle negative-stain reconstruction was ~25 Å, using an FSC cut-off value of 0.5. Representative top and side views of the 3D reconstructions are shown for reference.

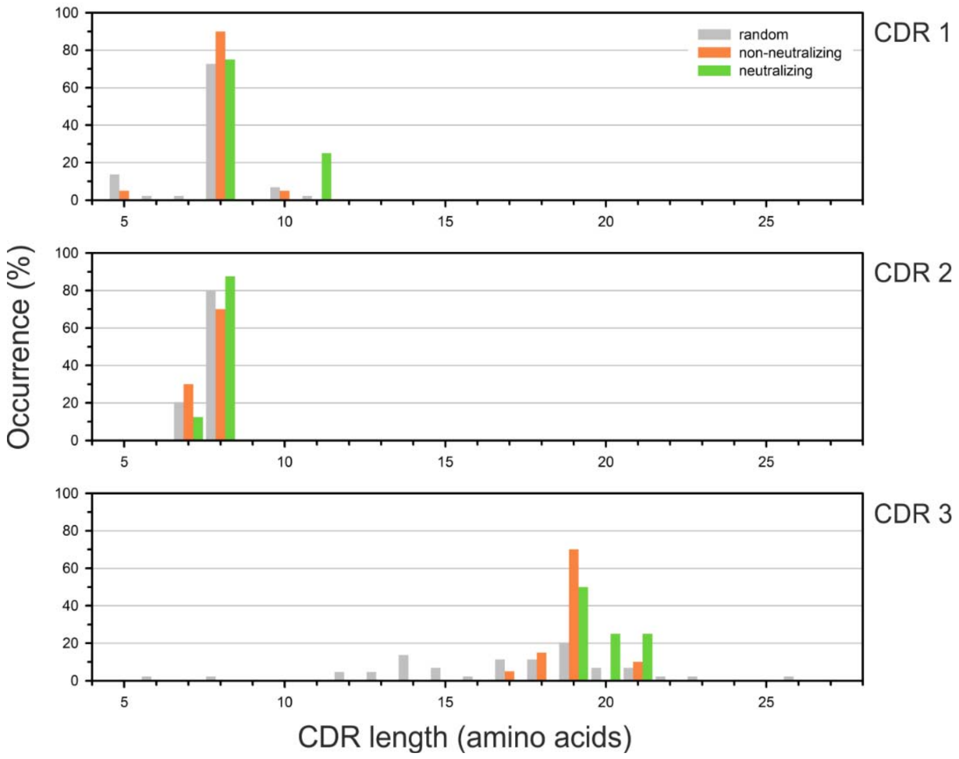

Supplementary figure S8: CDRs length distribution of dromedary VHHs. Top, middle, and bottom panel depict the amino acid length distribution of CDR 1, 2, and 3, respectively. CDR lengths were calculated using the IMGT definitions and involve the amino acid stretches from 27-38 (CDR 1), 56-65 (CDR 2), and 105-117 (CDR 3).[73] Groups labeled "non-neutralizing" and "neutralizing" were selected in this study and contain 20 and 8 unique sequences, respectively ("non-neutralizing": 43JI76, 43JI77, 43JI78, 43JI79, 43JI81, 43JI84, 43JI85, 43JI86, 43JI87, 43JI88, 65BH56, 65BH59, 65BI00, 65BI01, 65BI02, 65BI03, 65BI04, 65BI06, 65BI09, 65BI10; "neutralizing": VHH-A6, VHH-33, VHH-28, VHH-21, VHH-43, VHH-23, VHH-9, VHH-5). The group labeled "random" contains 44 unique sequences of dromedary VHHs selected against non-related targets and derived from the NCBI protein database (general identifiers: 158429305, 169791670, 194339183, 21730563, 21730569, 21730573, 268639693, 268639695, 304446109, 326787585, 33357355, 343197339, 38492670, 411031501, 411031503, 411031505, 411031507, 411031555, 71042017, 765365260, 765365262, 765365264, 765365266, 765365268, 765365270, 765365272, 765365274, 78101076, 816380365, 816380367, 816380369, 820947717, 820947719, 820947721, 93278623, 93278626, 93278628, 970842177, 984076509, 984076514, 984076519, 984076524, 984076529, and 9955227).

Supplementary Table S1: Neutralization (IC_{50}) of purified VHH-Fc on TZM-bl cells against a standard panel of pseudoviruses comprising different clades and neutralization sensitivities.

Pseudovirus	Tier	Clade	VHH-Fc 1	VHH-Fc 5	VHH-Fc 9	VHH-Fc 23	VHH-Fc 28	VHH-Fc 33	VHH-Fc 43	VHH-Fc A6
SF162	1A	B	> 50	1.82	0.035	0.13	0.25	0.030	0.85	0.029
Bal.26	1B	B	> 50	0.023	> 50	0.267	> 50	0.106	14.0	0.019
SS1196	1B	B	> 50	0.194	> 50	1.96	> 50	0.023	29.45	0.118
ZM197M	1B	C	> 50	0.856	28.1	> 50	2.17	> 50	> 50	0.126
MS208	2	A	1.01	> 50	> 50	> 50	11.8	> 50	> 50	> 50
398_F1_F6_20	2	A	> 50	> 50	0.205	> 50	0.277	> 50	> 50	> 50
X2278_C2_B6	2	B	> 50	3.20	10.5	> 50	> 50	0.063	> 50	0.075
REJO.4541	2	B	> 50	> 50	> 50	> 50	> 50	> 50	> 50	6.29
TRJO.4551	2	B	> 50	12.41	> 50	> 50	> 50	> 50	> 50	0.08
TRO.11	2	B	> 50	11.75	> 50	> 50	22.2	0.824	> 50	0.024
optC	n.d.	C	0.103	0.118	0.023	> 50	0.033	0.456	> 50	0.204
CAP45	2	C	> 50	24.55	> 50	> 50	> 50	> 50	> 50	> 50
CE1176_A3	2	C	5.33	49.33	0.155	> 50	0.058	1.14	> 50	0.244
CE703010217_B6	2	C	7.265	1.645	> 50	> 50	> 50	> 50	> 50	28.65
HIV_25710_2.43	2	C	21.0	> 50	0.098	6.69	0.020	0.065	2.26	0.335
246_F3_C10	2	AC	> 50	0.340	> 50	> 50	0.48	> 50	> 50	5.02
CNE55	2	CRF01/AE	7.04	> 50	1.3	> 50	0.011	> 50	> 50	0.603
X1632_S2	2	G	11.92	2.41	0.061	> 50	0.038	> 50	> 50	3.22
BJOX002000.03.2	2	CRF01/BC	> 50	> 50	5.075	> 50	> 50	> 50	> 50	> 50
CH119.10	2	CRF07/BC	> 50	> 50	> 50	> 50	1.24	49.25	> 50	> 50
CNE8	2	CRF01	> 50	> 50	> 50	> 50	49.5	> 50	> 50	35.5
MLV	-	-	> 50	> 50	> 50	> 50	> 50	> 50	> 50	> 50
**total**			**7/21**	**13/21**	**10/21**	**4/21**	**13/21**	**9/21**	**4/21**	**16/21**

IC50 [µg/ml]	> 50	10 -50	1 - 10	0.1 - 1	< 0.1

Supplementary Table S2: Determination of VHH epitopes via competitive ELISAs with known HIV-1 mAbs.

Target Env: optC gp140 SOSIP									
	HIV mAb	VHH 1	VHH 5*	VHH 9	VHH 23	VHH 28	VHH 33	VHH 43	VHH A6
CD4bs	F105	+++	-	+++	+++	++	-	+++	+++
	b12	+++	-	+++	+++	+++	-	+++	+++
	VRC01	-	-	++	-	-	-	-	-
	CD4-Fc	-	-	+++	++	-	-	+++	-
CD4i	17b	n.d.	n.d.	+++	+++	+++	n.d.	+++	+
gp41	246-D	-	-	-	-	-	-	-	-
Variable loops	697-30D	-	-	-	-	-	-	-	-
	PG9	-	-	-	-	-	-	-	-

Target Env: ZM197M gp140 SOSIP									
	HIV mAb	VHH 1***	VHH 5	VHH 9***	VHH 23**	VHH 28**	VHH 33***	VHH 43	VHH A6
CD4bs	F105	-	+++	-	+++	-	-	+++	+++
	b12	-	+++	-	-	-	-	+++	+++
	VRC01	-	+++	-	+	-	-	+++	+++
	CD4-Fc	-	++	-	-	-	-	+++	+++
CD4i	17b	n.d.	+++	n.d.	+	n.d.	n.d.	+++	+++
gp41	246-D	-	-	-	-	-	-	-	-
Variable loops	697-30D	-	-	-	-	-	-	-	+++
	PG9	-	-	-	-	-	-	-	-

ELISA plates were either coated with optC SOSIP (upper part) or with ZM197M SOSIP (lower part). 200 ng VHH were added, followed by the addition of the indicated HIV-1 mAbs, which were detected via a-human-HRP. Statistical differences in absorption (A450 – A650) between wells with VHH and wells with respective mAb only were determined using Two-way ANOVA and Bonferroni posttests. +++: $P < 0.001$, ++: $P < 0.01$, +: $P < 0.05$, - : $P > 0.05$. */**/***: weak to very weak binding to indicated Env.

Supplementary Table S3: Data collection and refinement statistics.

	VHH5	VHH28
Beamline	SSRL 12-2	SSRL 12-2
Wavelength (Å)	0.97946	0.97946
Resolution (Å)[a]	55.3-2.30 (2.36-2.30)	28.4-1.15 (1.17-1.15)
Space group	C2	$C222_1$
Unit cell (Å, °)	89.41, 48.48, 60.89 90.00, 114.67, 90.00	56.79, 76.94, 69.66 90.00, 90.00, 90.00
Total reflections	67,799 (5110)	319,018 (11,285)
Unique reflections	10,575 (774)	53,677 (2625)
Multiplicity	6.4 (6.6)	5.9 (4.3)
Completeness	99.2, (100.0)	99.5 (97.9)
Mean (I)/(σ_1)	8.6 (3.5)	17.2 (1.3)
R_{merge}[b](%)	19.9 (65.9)	9.8 (154.3)
R_{meas}[c] (%)	21.7 (71.7)	10.7 (175.8)
R_{pim}[d] (%)	8.5 (27.8)	4.3 (82.5)
$CC_{1/2}$[e]	99.0 (92.7)	88.0 (45.0)
R_{work} (%)	20.1 (27.4)	14.5 (32.1)
R_{free} (%)	26.1 (32.9)	17.0 (32.7)
#reflections used in refinement (work/free)	10,081/493	50,931/2706
#protein atoms	1911	1123
#water molecules	42	168
#protein residues	253	127
RMS (bonds)	0.016	0.022
RMS (angles)	1.83	1.76
Ramachandran favored, allowed, outliers	97.2, 2.8, 0.0	98.0, 2.0, 0.0
Clashscore[f]	3.0	6.8
Wilson B (Å^2)	21.8	12.4
Average B (Å^2)	23.2	20.1
Protein	23.1	19.2
Solvent	55.9	33.0

[a]Numbers in parentheses are for highest resolution shell
[b]$R_{merge} = \Sigma_{hkl} \Sigma_{i=1,n}|I_i(hkl)- \langle I(hkl)\rangle| / \Sigma_{hkl} \Sigma_{i=1,n} I_i(hkl)$
[c]$R_{meas} = \Sigma_{hkl} \sqrt{(n/n-1)} \Sigma_{i=1,n}|I_i(hkl)- \langle I(hkl)\rangle| / \Sigma_{hkl} \Sigma_{i=1,n} I_i(hkl)$
[d]$R_{pim} = \Sigma_{hkl} \sqrt{(1/n-1)} \Sigma_{i=1,n}|I_i(hkl)- \langle I(hkl)\rangle| / \Sigma_{hkl} \Sigma_{i=1,n} I_i(hkl)$
[e]$CC_{1/2}$ = Pearson Correlation Coefficient between two random half datasets
[f]Number of unfavorable all-atom steric overlaps ≥ 0.4Å per 1000 atoms
